# Machine learning-based seasonal SMAP soil moisture retrieval integrating MODIS drought indices: A case study of the Wujiang River Basin

**DOI:** 10.1371/journal.pone.0351643

**Published:** 2026-06-22

**Authors:** Ju Zhao, Hanyu Lu, Pengfei Qu, Yongyi Yuan

**Affiliations:** 1 College of Big Data and Information Engineering, Guizhou University, Guiyang, Guizhou, China; 2 Bijie City Artificial Intelligence Application Innovation Talent Team, Guizhou University of Engineering Science, Bijie, Guizhou, China; Tishk International University, IRAQ

## Abstract

Soil moisture (SM) is a critical regulator of energy and water exchange between the land and atmosphere, yet its accurate retrieval in complex Karst terrains remains challenging due to extreme surface heterogeneity and intricate hydro-thermal coupling. Traditional unified modeling approaches often struggle to capture the seasonally varying, non-linear relationships between remote sensing signals and moisture dynamics in fragmented landscapes. To address these limitations, this study developed a seasonally decoupled machine learning framework, utilizing long-term data from 2019 to 2024, to enhance the spatial representativeness of 9 km SMAP products in the Wujiang River Basin. By integrating 14 MODIS-derived drought indicators with static topographic factors, we constructed differentiated seasonal input sets to account for the “signal decoupling” potentially caused by intense precipitation pulses and phenological shifts. Five algorithms, including RBFNN, SVM, RF, XGBoost, and CatBoost, were systematically evaluated under the Optuna optimization framework. Quantitative results suggest that the CatBoost-based decoupled model achieved an improved balance of accuracy and robustness, with $R^{\text{2}}$ values reaching 0.537 and 0.572 in spring and autumn, respectively, showing certain advantages over traditional baseline models. Importantly, SHAP-based attribution analysis identifies statistical patterns consistent with a transition in hydrological behaviors, indicating that the model’s decision logic shifts from a ‘topography-driven’ importance in winter to a ‘hydro-thermal-driven’ dominance in summer. This seasonally adaptive approach contributes to mitigating systematic biases in satellite products and provides a potential methodological reference for drought monitoring and water resource management in complex mountainous environments.

## Introduction

Soil moisture (SM) serves as a pivotal medium linking the atmosphere, hydrosphere, biosphere, and lithosphere, and is recognized as a core variable within terrestrial eco-hydrological-climatic systems [1]. The availability of water resources in the vegetation root zone is directly determined by SM, which further regulates the intensity of plant photosynthesis and respiration. Consequently, the partitioning patterns of surface latent and sensible heat fluxes are influenced by SM, playing a fundamental role in crop growth, yield formation, and ecosystem stability [2]. Particularly in arid and semi-arid regions, the sensitivity of agriculture to drought stress and the success of vegetation restoration processes are largely dictated by soil moisture status. Thus, SM remains a critical factor constraining ecological reconstruction and the management of land degradation [3].

With the widespread application of soil moisture retrieval techniques, systematic research tailored to diverse application scenarios and the continuous optimization of models have become increasingly essential. Conventional approaches for acquiring soil moisture data, including ground-based observations and numerical simulations [4], are frequently constrained by limited spatial coverage and insufficient representativeness, making it difficult to meet the requirements for large-scale and multi-temporal monitoring. In contrast, remote sensing (RS) technology has emerged as an effective alternative for soil moisture research [5–8], owing to its extensive spatial coverage, high temporal efficiency, and superior spatial continuity. Remote sensing data are primarily categorized into microwave and optical remote sensing, based on the different sensing bands and techniques employed. All-weather and all-day monitoring capabilities are offered by microwave remote sensing, with the ability to penetrate cloud cover and atmospheric interference, rendering it indispensable for soil moisture monitoring [9–10]. Key land surface parameters, such as vegetation indices and land surface temperature (LST), are efficiently acquired through optical remote sensing via visible and thermal infrared bands. These data are highly intuitive, accessible, and easily processed, supported by mature interpretation tools and extensive historical datasets [11]. In view of these complementary strengths, recent years have seen a transition toward more integrated monitoring frameworks that combine physical principles with satellite observations to overcome the inherent limitations of single-source data [12].

In recent years, drought indices have become vital tools for remote sensing-based soil moisture retrieval, and their ability to effectively characterize the spatiotemporal variations of SM has been validated across numerous studies. In a study by Xie and Fan [13], time series of the Normalized Difference Vegetation Index (NDVI) and LST from the Moderate Resolution Imaging Spectroradiometer (MODIS) were utilized, where it was found that data reconstruction significantly enhances the monitoring performance of drought indices. MODIS data were leveraged by Wang et al. [14] to construct the Vegetation Supply Water Index (VSWI), through which the spatiotemporal dynamics of spring drought in Yunnan were successfully monitored, confirming the regional applicability of the VSWI. A comprehensive drought index—the Surface Water Capacity Index (SWCI)—was developed by Li et al. [15] through the integration of multiple hydro-meteorological elements, systematically revealing future drought trends in the Ili River Basin. Furthermore, the Normalized Difference Water Index (NDWI) was combined with soil loss models by Rendana et al. [16] to analyze the spatial correlation between drought and soil erosion. The Normalized Multi-band Drought Index (NMDI), proposed by Wang and Qu [17], utilizes combinations of near-infrared and shortwave infrared bands to enhance monitoring capabilities for both soil moisture and vegetation water content, enabling the differentiation of drought types under various vegetation covers. Additionally, the Humidity-calibrated Drought Condition Index (HeDCI) was constructed by Li et al. [18] based on soil moisture calibration, revealing the spatiotemporal evolution of agricultural drought in Weihai, China. Recent improvements to the Temperature Vegetation Dryness Index (TVDI) were achieved by Huang et al. [19] through the introduction of dynamic thresholds, attaining higher precision in the Huai River Basin. The weight allocation of the Vegetation Condition Index (VCI) and Temperature Condition Index (TCI) was optimized by Zeng et al. [20], and the self-calibrating Palmer Drought Severity Index (sc-PDSI) was integrated to construct a global high-resolution Vegetation Health Index (VHI) dataset, significantly improving detection efficiency. It was demonstrated by Sun et al. [21] that global vegetation greening increases potential evapotranspiration, leading to intensified drought in over 55% of vegetated areas through a comparison of Standardized Precipitation Evapotranspiration Index (SPEI) simulations. Nevertheless, the sensitivity of different remote sensing drought indices to vegetation cover is subject to variation, and monitoring capacities are observed to fluctuate with environmental conditions, which affects the universality and accuracy of drought assessments [22]. Given that soil moisture is a non-linear coupled system influenced by meteorology, vegetation, and soil texture, its complex dynamics are often difficult to fully capture with a single drought index. Therefore, the development of multi-parameter non-linear integration methods that fuse multi-source remote sensing data with ground observations has become a primary research direction [23]. Such methods, utilizing modeling frameworks like data assimilation and machine learning, can effectively integrate multi-dimensional information to systematically enhance the representation and prediction of drought processes.

Consequent to advancements in machine learning (ML) algorithms, robust computational tools capable of handling both linear and non-linear relationships—such as eXtreme Gradient Boosting (XGBoost), Categorical Boosting (CatBoost), and Radial Basis Function Neural Networks (RBFNN)—have been extensively implemented in remote sensing [24–28]. Although excellent performance is exhibited by these models across various tasks, their efficacy is often observed to vary depending on the application scenario and data characteristics. For instance, it was shown by Nguyen et al. [29] that Extreme Gradient Boosting Regression combined with a Genetic Algorithm (XGBR-GA) outperformed Random Forest Regression (RFR), Support Vector Machines (SVM), and CatBoost in soil moisture estimation. RBFNN was utilized by Wang et al. [30] to effectively fit the non-linear relationships among drought monitoring parameters, successfully extending point-scale station data to regional scales. Additionally, a systematic comparison between SVM and Random Forest (RF) algorithms was conducted by Lakra et al. [31]; by fusing Sentinel-1 Synthetic Aperture Radar (SAR) backscatter with radar vegetation indices, a soil moisture estimation model was constructed, further validating the robustness of RF. These studies indicate that unique advantages are possessed by different ML algorithms under varying regional conditions, necessitating selection based on specific application requirements. Building on this, contemporary research has further explored the synergy between multi-source data, such as SAR and optical imagery, to enhance retrieval robustness in complex landscapes—a trend that underscores the importance of information fusion in modern machine learning applications [32].

Considering the highly fragmented terrain and pronounced seasonal heterogeneity of hydrothermal processes in Karst regions [33], conventional unified modeling frameworks often struggle to accurately capture the intricate spatiotemporal evolution of soil moisture. To address these limitations, this study integrates 14 multi-source remote sensing features, encompassing land surface temperature (LST), evapotranspiration (ET), vegetation indices (e.g., NDVI, LAI), and multiple moisture deficit and drought monitoring indicators (e.g., TVDI, VSDI). The primary innovation of this work is the development of a seasonally decoupled retrieval framework. By conducting experiments in the Wujiang River Basin, a typical Karst geomorphology region, this study aims to: (1) employ Pearson correlation analysis to reveal the seasonal sensitivity variations of multi-source remote sensing factors to soil moisture (SM), and subsequently construct seasonal dynamic feature input sets to address the complex hydrothermal constraints of the basin; (2) evaluate and compare the robustness and generalization capabilities of five machine learning models in processing highly heterogeneous nonlinear surface data, thereby verifying the effectiveness of the seasonally decoupled retrieval framework in enhancing cross-seasonal predictive performance; and (3) incorporate SHAP attribution analysis to elucidate the internal physical mechanisms through which multi-source feature fusion improves the spatiotemporal representativeness of SMAP products at the basin scale. This research not only provides a scientific basis and analytical reference for monitoring drought evolution in the Wujiang River Basin but also offers methodological insights for interpretability studies and optimization of remote sensing retrieval in complex environments.

## Materials and methods

This section provides a comprehensive description of the geographical setting of the Wujiang River Basin and the multi-source remote sensing datasets utilized in this study.

### Study area overview

**Fig 1:**
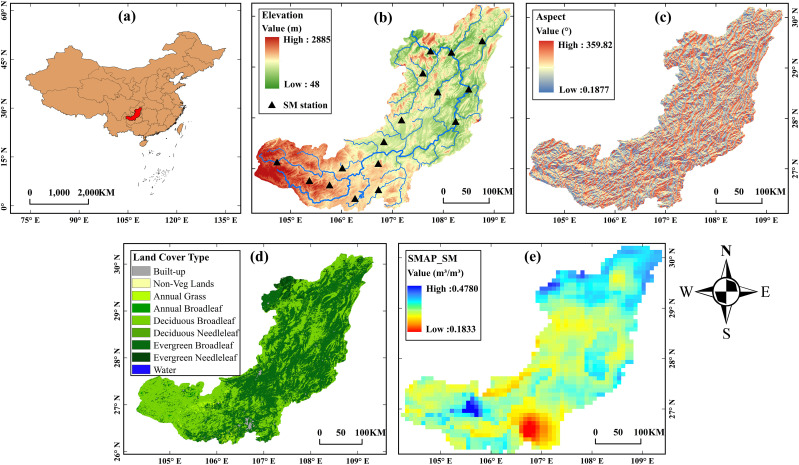
Overview of the study area. **(a)** Location of the Wujiang River Basin in China; **(b)** Elevation and distribution of monitoring stations; **(c)** Slope aspect distribution; **(d)** Land cover types; **(e)** SMAP soil moisture grid data.

The Wujiang River Basin, located in Southwest China and encompassing most of Guizhou Province and parts of Chongqing Municipality, was selected as the study area. The geographic extent of the basin is defined by coordinates ranging from 104°18′ to 109°22′ E and 26°07′ to 30°22′ N. A complex and diverse topography is exhibited across the region, which is primarily characterized by plateau mountains, hills, and canyon landscapes (Fig 1(a)). A significant elevation gradient, ranging from 48 m to 2,885 m, is observed within the region (Fig 1(b)), resulting in the formation of a prominent vertical terrain pattern. Sharp variations in slope aspects (Fig 1(c)) are further induced by this complex topography; the substantial differences in hydrothermal conditions between sunny and shady slopes profoundly influence local microclimates and eco-hydrological processes. The study area is situated within a subtropical humid monsoon climate zone, where abundant precipitation and four distinct seasons are prevalent. A pronounced redistribution of hydrothermal resources is facilitated by the intricate terrain, leading to a high degree of spatial heterogeneity in climatic features. The annual sunshine duration is estimated at approximately 1,100–1,400 hours, with climatic conditions varying according to latitude, topography, altitude, and atmospheric circulation. Humid and mild conditions are maintained year-round in the upstream region, which is influenced by southwest air currents; this area is characterized by an average annual temperature of approximately 14 °C, a frost-free period of 250–300 days, and annual precipitation of roughly 1,000 mm. In contrast, a warmer climate is experienced within the middle and downstream areas, where average annual temperatures range from 16 °C–18 °C, the frost-free period exceeds 300 days, and total annual precipitation reaches 1,200–1,400 mm. The concentration of rainfall is observed primarily from May to September, representing over 70% of the total annual precipitation.

The administrative boundaries and base maps utilized in Fig 1 and Fig 3 were adapted from the standard map (No. GS(2019)1822) provided by the Map Technical Review Center, Ministry of Natural Resources of China (http://bzdt.ch.mnr.gov.cn), under a CC BY license with permission. All thematic maps were generated using the open-source Geographic Information System, QGIS (https://www.qgis.org), under a CC BY license. The primary research layers, including the Digital Elevation Model (DEM), Land Cover types, and SMAP soil moisture data, were derived from the authors’ processed datasets or publicly available domain sources.

**Fig 2:**
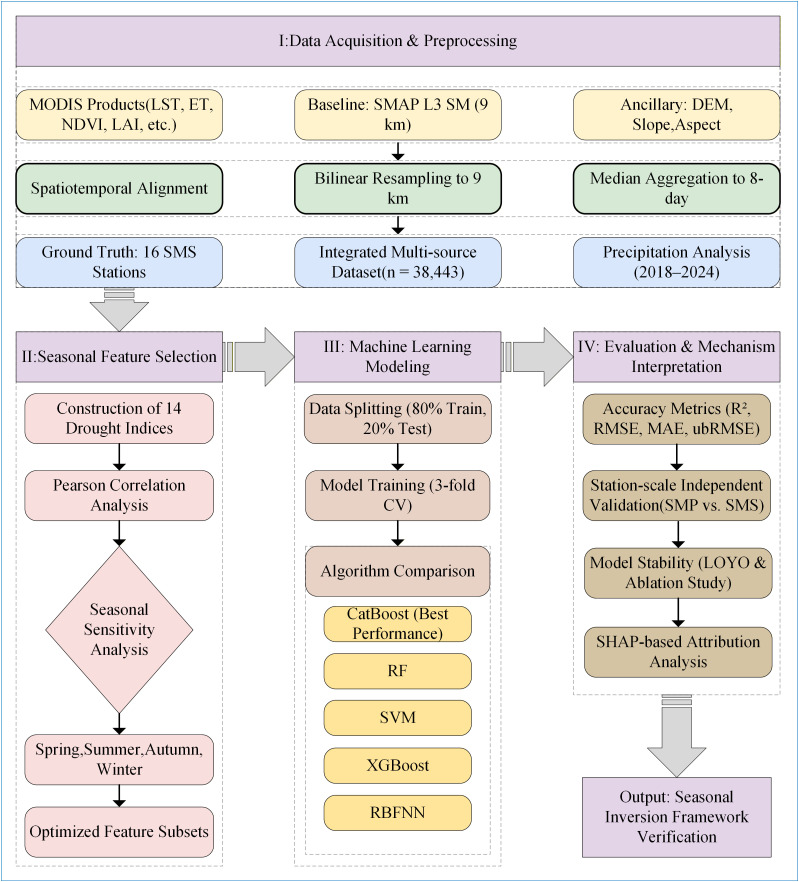
Research framework for soil moisture retrieval model construction.

### Research framework

This study developed a seasonally decoupled soil moisture (SM) retrieval framework (Fig 2), which aims to address the challenges posed by high surface heterogeneity and pronounced seasonal variations in hydrothermal combinations within the Wujiang River Basin. The research workflow consists of four primary stages. First, in the data acquisition and feature engineering stage, multi-source remote sensing data for the Wujiang River Basin from 2019 to 2024 were collected. Fourteen MODIS-derived characteristic parameters—encompassing vegetation (NDVI, LAI), moisture (NDWI, VSDI), temperature (LST, TVDI), composite drought indices (SWCI, VSWI), and evapotranspiration (ET)—were extracted, alongside topographic factors (DEM, slope, and aspect) to characterize the geographic baseline. To ensure spatial consistency, all surface feature parameters were aligned to a 9 km spatial resolution using bilinear resampling and median aggregation to match the pixel size of the SMAP surface SM products, resulting in a spatiotemporally matched sample library at an 8-day scale. Subsequently, in the feature optimization stage, Pearson correlation analysis was employed to evaluate the association strength between each parameter and the SM labels across different seasons. By revealing the dynamic evolution of dominant factors, differentiated sensitive feature combinations were constructed for seasonal modeling. During the model construction and training stage, a seasonally independent modeling strategy was implemented to accommodate the complex hydrothermal dynamics of the Karst environment. Five algorithms (RBFNN, SVM, RF, CatBoost, and XGBoost) were systematically compared for their performance in establishing nonlinear mapping relationships, with the Optuna framework utilized for hyperparameter optimization to enhance model robustness under extreme climatic pulses. Finally, in the multi-level evaluation and attribution stage, in addition to standard accuracy metrics ($R^{\text{2}}$, RMSE, MAE, and ubRMSE), Leave-one-year-out (LOYO) cross-validation and feature ablation experiments were introduced to assess the spatiotemporal generalization and stability of the models. Independent validation was also conducted using data from 16 ground stations (SMS) to explore the effectiveness of integrating multi-source auxiliary information in mitigating representation errors of the original satellite products. Furthermore, SHAP-based attribution analysis was performed to interpret the model decision logic from a physical mechanism perspective, elucidating the varying contributions of environmental factors across seasons and achieving transparency and interpretability in the retrieval process.

### Data sources

This section details the geographical origin and technical specifications of the datasets used for seasonal soil moisture retrieval. The following subsections describe the acquisition of in-situ observations and the spatiotemporal synchronization of multi-source remote sensing imagery.

#### In-situ soil moisture data.

Soil relative humidity data utilized in this research were sourced from the China Meteorological Data Service Center (http://data.cma.cn). Observation records are provided within this dataset at a daily temporal resolution, encompassing parameters such as crop growth status, farmland soil moisture, and relative soil moisture at various depths as recorded by specialized monitoring stations. For the period spanning 2019–2020, 16 stations characterized by high data integrity were selected within the study area (Fig 1(b)), ensuring the acquisition of complete observation records over two consecutive years.

#### Remote sensing imagery data.

Multi-source remote sensing data spanning from December 2018 to November 2024 were integrated in this research, encompassing the entirety of the Wujiang River Basin (tiles H26V06, H27V06, and H27V07). Included within the dataset were various MODIS products (MOD16A2GF, MOD11A2, MOD09A1, and MOD15A2H) acquired from the official NASA website (https://www.earthdata.nasa.gov), SMAP (Soil Moisture Active Passive) surface soil moisture products obtained via Google Earth Engine (https://earthengine.google.com), and the SRTM 90 m Digital Elevation Model (DEM) provided by the Consortium for Spatial Information (CGIAR-CSI) (http://srtm.csi.cgiar.org). To reconcile the disparate native resolutions and temporal frequencies of these products—specifically MOD16A2GF (500 m, 8 days), MOD11A2 (1 km, 8 days), MOD09A1 (500 m, 8 days), MOD15A2H (500 m, 8 days), SMAP (9 km, 1 day), and SRTM DEM (90 m)—a systematic preprocessing workflow was implemented to ensure spatiotemporal synchronization. Specifically, a spatial resampling of all MODIS indices and DEM data to a 9 km resolution was executed utilizing the bilinear interpolation method. Furthermore, the synthesis of daily SMAP data into 8-day intervals was accomplished via median aggregation, culminating in the construction of a multi-source fusion dataset optimized for drought index derivation and model-based soil moisture retrieval.

### Methodology

In this section, the technical details of the seasonally decoupled framework, the selection of machine learning algorithms, and the evaluation metrics are elaborated.

#### Construction of MODIS optical indices.

Extensive global research on soil moisture retrieval and drought monitoring utilizing soil moisture observational data has been conducted by various scholars [34–41]. Founded upon these precedents, a comprehensive drought monitoring dataset was constructed in this research through the integration of multi-source parameters, encompassing variables such as soil moisture, vegetation indices, water indices, temperature indices, composite indices, and evapotranspiration (ET). 14 principal remote sensing drought indicators, including NDVI, LAI, and LST, were identified and selected for the present study. The mathematical formulations for each respective index are detailed in Table 1.

**Table 1:** Remote sensing parameters and calculation methods.

Remote Sensing Parameters	Drought Indices	Calculation Formula	Literature
Vegetation Indices	NDVI	$\text{NDVI}=\frac{b_{\text{2}}-b_{\text{1}}}{b_{\text{2}}+b_{\text{1}}}$	[42]
	VCI	$VCI=\frac{NDVI-NDVI_{min}}{NDVI_{max}-NDVI_{min}}$	[43]
	VHI	VHI=a×VCI+(1−a)×TCI\hspace{1em}(a=0.5)	[44]
	LAI		[45]
Moisture Indices	NDWI	$\text{NDWI}=\frac{b_{\text{2}}-b_{\text{4}}}{b_{\text{2}}+b_{\text{4}}}$	[46]
	NMDI	$\text{NMDI}=\frac{b_{\text{2}}-(b_{\text{6}}-b_{\text{7}})}{b_{\text{2}}+(b_{\text{6}}-b_{\text{7}})}$	
	VSDI1	$\text{VSDI1}=1-\left[\left(b_{\text{6}}-b_{\text{3}}\right)+\left(b_{\text{1}}-b_{\text{3}}\right)\right]$	[47]
	VSDI2	$\text{VSDI2}=1-\left[\left(b_{\text{7}}-b_{\text{3}}\right)+\left(b_{\text{1}}-b_{\text{3}}\right)\right]$	
Temperature Indices	TCI	$\text{TCI}=\frac{{\text{LST}}_{max}-\text{LST}}{{\text{LST}}_{max}-{\text{LST}}_{min}}$	[48]
	TVDI	$\text{TVDI}=\frac{\text{LST}-{\text{LST}}_{\left(min\right)}\left(\text{NDVI}\right)}{{\text{LST}}_{\left(max\right)}\left(\text{NDVI}\right)-{\text{LST}}_{\left(min\right)}\left(\text{NDVI}\right)}$ ${\text{LST}}_{\left(max\right)}=a_{\text{1}}+c_{\text{1}}{\text{NDVI,LST}}_{\left(min\right)}=a_{\text{2}}+c_{\text{2}}\text{NDVI}$	[49]
	LST		[50]
Composite Indices	SWCI	$\text{SWCI}=\frac{b_{\text{6}}-b_{\text{7}}}{b_{\text{6}}+b_{\text{7}}}$	[51]
	VSWI	$\text{VSWI}=\frac{\text{NDVI}}{\text{LST}}$	[52]
Evapotranspiration	ET		[53]
Soil Moisture Data	SM		

The spectral configurations for the MOD09A1 product consist of bands $b_{\text{1}}$(620–670 nm), $b_{\text{2}}$(841–876 nm), $b_{\text{3}}$(459–479 nm), $b_{\text{4}}$(545–565 nm), $b_{\text{6}}$(1628–1652 nm), and $b_{\text{7}}$(2105–2155 nm). In the present research, LST is utilized to denote land surface temperature, whereas $LST_{max}$ and $LST_{min}$ represent the multi-year maximum and minimum land surface temperatures observed for the same period. Similarly, $NDVI_{max}$ and $NDVI_{min}$ are defined as the maximum and minimum NDVI records for the corresponding timeframe. Regarding the Temperature Vegetation Dryness Index TVDI, the dry and wet edge equations are defined by $LST_{\left(max\right)}$ and $LST_{\left(min\right)}$, where $a_{\text{1}}$, $c_{\text{1}}$, $a_{\text{2}}$, and $\;c_{\text{2}}$ serve as the regression coefficients to be determined. Furthermore, a weighting factor  (a) of 0.5 is implemented in the Vegetation Health Index VHI calculation. Finally, ET and SM are utilized to represent evapotranspiration and the SMAP satellite soil moisture data, respectively.

#### Feature selection via pearson correlation analysis.

Pearson correlation analysis is recognized as a conventional covariance-based statistical method and is utilized to quantify both the strength and direction of the linear association between two continuous variables, where the Pearson correlation coefficient r serves as the primary metric [54]. The determination of this coefficient is achieved by calculating the ratio between the covariance of the two variables and the respective product of their standard deviations; the mathematical formulation is provided as follows:


$$\begin{array}{c} r=\frac{\sum_{i=1}^{n}(X_{i}-\overset{―}{X})(Y_{i}-\overset{―}{Y})}{\sqrt{\sum_{i=1}^{n}(X_{i}-\overset{―}{X}{)}^{\text{2}}}\sqrt{\sum_{i=1}^{n}(Y_{i}-\overset{―}{Y}{)}^{\text{2}}}} \end{array}$$ (1)

Within this framework, the i-th observed values of the two variables are represented by $X_{\text{i}}$ and $Y_{i}$ while their respective mean values are signified by $\overset{―}{X}$and $\overset{―}{Y}$，with n denoting the total sample size. The magnitude of the correlation coefficient r spans from –1–1; specifically, r=1 denotes a perfect positive correlation, r=−1 represents a perfect negative correlation, and r=0 implies the complete absence of a linear relationship. Through the evaluation of statistical significance and the absolute value of |r|,the linear relationship between variables is determined, thereby facilitating the effective quantification of associations between independent and dependent variables. This analysis establishes a critical foundation for subsequent modeling and the strategic selection of features.

In this study, Pearson correlation analysis was implemented as a preliminary screening tool to identify prominent linear associations, with the intent of reducing dimensional redundancy and irrelevant noise. While this approach effectively refines the initial feature set, the potential non-linear dependencies between environmental indicators and soil moisture are subsequently addressed through machine learning algorithms. Given their capacity for resolving intricate hydro-thermal coupling in Karst terrains, these models are expected to effectively incorporate both linear and complex non-linear interactions into the proposed retrieval framework.

#### Machine learning algorithms.

The Radial Basis Function Neural Network (RBFNN), initially proposed by Broomhead and Lowe [55], is recognized as an established feedforward neural network model characterized by exceptional performance and convergence speeds in function approximation, pattern recognition, and nonlinear regression. Due to its architectural simplicity and global approximation capabilities, this model has been extensively employed in time-series prediction, system modeling, and classification tasks. The widespread adoption of the RBFNN is primarily attributed to its rapid training speed, structural transparency, and inherent robustness against converging to local optima. Complex nonlinear mappings can be achieved with a limited number of hidden layer nodes, while clear mathematical interpretability is maintained. The fitting and generalization performance of the model are directly influenced by key hyperparameters, including the centers and widths (standard deviations) of the radial basis functions and the weights of the output layer.

The Random Forest (RF) algorithm, introduced by Breiman [56], represents an ensemble learning method that enhances predictive accuracy via the integration of multiple decision trees. Superior generalization capabilities and robustness are exhibited by this model in both classification and regression tasks; furthermore, RF is characterized by its insensitivity to noisy data and high resistance to overfitting. The extensive implementation of RF in scientific research is primarily attributed to the mechanisms of bootstrap sampling and random feature selection. These mechanisms effectively attenuate model variance and optimize overall predictive performance while preserving robust interpretability and stability when processing high-dimensional datasets. The fitting efficacy and generalization ability of the model are collectively determined by key hyperparameters, including the number of trees, maximum depth, the number of features selected, and the node-splitting criteria.

The eXtreme Gradient Boosting (XGBoost) algorithm, introduced by Chen and Guestrin [57], is a high-efficiency gradient-boosted decision tree framework that exhibits superior predictive performance and generalization capability. It is further characterized by high scalability and computational efficiency. The widespread implementation of XGBoost is primarily attributed to the incorporation of a regularization term within the objective function to meticulously control model complexity. This is coupled with advanced computational techniques, such as parallelization, out-of-core computation, and cache optimization, which significantly optimize training speed and resource utilization. The predictive precision and generalization capability of the model are directly influenced by key hyperparameters, including the learning rate, maximum tree depth, number of trees, and minimum split loss.

The Categorical Boosting (CatBoost) algorithm, introduced by Prokhorenkova et al. [58], is an advanced gradient boosting decision tree (GBDT) framework applicable to classification, regression, and ranking tasks, demonstrating particular efficacy in managing categorical features. Gradient bias and overfitting issues are effectively mitigated through the employment of an ordered boosting strategy and a symmetric tree structure. More recently, this framework has been successfully implemented within environmental modeling domains, such as soil parameter estimation. CatBoost is distinguished by its high training efficiency and inherent robustness, even when utilizing default parameter configurations. The predictive performance of the model is governed by primary hyperparameters, including the learning rate, maximum tree depth, and total number of iterations.

The Support Vector Machine (SVM), initially introduced by Cortes and Vapnik [59], is a supervised learning method grounded in statistical learning theory and the kernel trick. Effective learning is achieved through the identification of the maximum margin hyperplane, while nonlinear complexities are addressed via the implementation of kernel functions, resulting in superior generalization performance. Nevertheless, the model may exhibit sensitivity toward datasets containing substantial noise or significant categorical overlap. The performance of the SVM is primarily governed by key hyperparameters, including the regularization parameter, the selection of the kernel function, and associated kernel parameters. Frequently implemented kernel functions encompass linear, polynomial, radial basis function (RBF), and sigmoid variants.

#### Hyperparameter optimization based on the Optuna framework.

To ensure objectivity and fairness in the performance comparison among various machine learning models, the Optuna framework was introduced for automated hyperparameter optimization. This framework employs the Tree-structured Parzen Estimator (TPE) algorithm [60] to efficiently search for global optimal parameters within a complex high-dimensional space, utilizing the Root Mean Square Error (RMSE) of the test set as the objective function. The TPE algorithm partitions historical evaluation results into two groups—well-performing and underperforming—based on a quantile threshold (y*), and constructs probabilistic density surrogate models for the hyperparameters respectively:


$$\begin{array}{c} p(x|y)=\left\{ \begin{array}{cc} @ll@l\left(x\right), & \text{if}y<y^{*}\\ g\left(x\right), & \text{if}y\geq y^{*} \end{array}\right. \end{array}$$ (2)

Where x represents a given hyperparameter combination; y is the corresponding model error; and l(x) and  g(x) denote the probability density functions when the error is less than (well-performing) and greater than or equal to (underperforming) the threshold y*, respectively. On this basis, the TPE algorithm identifies the maximum Expected Improvement (EI) by maximizing the probability density ratio l(x)/g(x). This mechanism effectively balances the exploration of unknown parameter spaces with the exploitation of known high-quality parameters, thereby guiding each retrieval model to converge rapidly toward the optimal configuration at a lower computational cost.

#### Model interpretability analysis based on SHAP.

To address the inherent limitations of machine learning models in revealing the physical associations within surface soil moisture retrieval, this study introduces the SHAP (SHapley Additive exPlanations) attribution framework [61]. Grounded in cooperative game theory, this method assigns Shapley values by calculating the average marginal contribution of each feature across all possible permutations. Compared with traditional Gini importance, SHAP offers local accuracy and consistency, which helps mitigate attribution biases caused by collinearity among features. The additive feature attribution model is formulated as follows:


$$\begin{array}{c} g\left(z^{\prime }\right)=\phi_{\text{0}}+\sum_{i=1}^{M}\phi_{i}{z^{\prime }}_{i} \end{array}$$ (3)

Where $g\left(z^{\prime }\right)$ denotes the explanation model; M is the total number of features (M=14); $\phi_{\text{0}}$ represents the base value of the model prediction; $\phi_{i}$ is the contribution value (SHAP value) of the i-th feature; and ${z^{\prime }}_{i}\in \{0,1{\}}^{M}$ indicates the observation status of feature i (where 1 signifies observable and 0 signifies missing). A value of $\phi_{i}>0$ suggests a potential positive driving effect on the retrieval results, while $\phi_{i}<0$ implies an inhibitory effect.

In this study, an attribution algorithm optimized for tree-based structures is employed to construct seasonal summary plots comprising global feature importance bars and SHAP beeswarm plots. This approach not only provides a preliminary ranking of the average contributions of the 14 indicators from a global perspective but also explores the potential nonlinear driving directions and influence intensities of feature values by observing the color distribution and horizontal displacement of sample points. This method contributes to the analysis of seasonal variations in underlying surface factors within the Karst mountainous region and aims to provide a reference for understanding the hydro-physical logic underlying data-driven models.

#### Validation strategy and generalization assessment.

To evaluate the reliability of the retrieval models, a dual-validation framework was constructed. Initially, a conventional random-split strategy (with a training-to-test ratio of 8:2) was employed to conduct a preliminary assessment of the models’ fitting capability within the known sample space. Considering the significant inter-annual climatic fluctuations in the Karst region, conventional random splitting may exhibit biases when evaluating the models’ extrinsic extrapolation performance across different years.

Consequently, this study further introduced a Leave-One-Year-Out (LOYO) strategy [62]. Utilizing the research sequence from 2019 to 2024, an independent year was sequentially reserved as the validation set, while the Optuna framework was integrated within each cycle for dynamic hyperparameter optimization. This approach seeks to preliminarily simulate the models’ adaptive capacity to unseen meteorological backgrounds. Furthermore, while the LOYO strategy effectively addresses temporal overfitting by isolating annual cycles, it is important to acknowledge that residual spatial dependence may persist within the dataset, as neighboring pixels in fragmented Karst terrains are not entirely independent. Future refinements could explore more advanced spatial-temporal buffering techniques to further decouple these localized dependencies. In the statistical processing, the arithmetic mean of the precision indicators from the K validation cycles (${\text{Score}}_{\text{LOYO}}$) was calculated to evaluate the applicability of the seasonal retrieval framework under the complex underlying surfaces of the Wujiang River Basin:


$$\begin{array}{c} {\text{Score}}_{LOYO}=\frac{\text{1}}{K}\sum_{k=1}^{K}{\text{Score}}_{k} \end{array}$$ (4)

where ${\text{Score}}_{\text{LOYO}}$ represents the average precision metric after LOYO validation; K denotes the total number of typical years involved in the validation (K=6 in this study); k is the index of the current validation cycle; and ${\text{Score}}_{k}$ is the predictive accuracy achieved by the model when the k-th year serves as the independent validation set. This method aims to provide a more objective assessment of model robustness against inter-annual variability and strives to offer a methodological reference for long-term soil moisture monitoring in this region.

#### Model performance evaluation.

To quantitatively evaluate the predictive performance of each model during the aforementioned validation processes, four statistical metrics widely utilized in the field of hydrological remote sensing were selected: the Coefficient of Determination ($R^{\text{2}}$), Root Mean Square Error (RMSE), Mean Absolute Error (MAE), and Unbiased Root Mean Square Error (ubRMSE). The calculation formulas for these metrics are as follows:


$$\begin{array}{c} R^{\text{2}}=1-\frac{\sum_{\text{i}=1}^{n}(X_{i}-Y_{i}{)}^{\text{2}}}{\sum_{\text{i}=1}^{n}(X_{i}-{\overset{―}{X}}_{i}{)}^{\text{2}}} \end{array}$$ (5)

$$\begin{array}{c} RMSE=\sqrt{\frac{\text{1}}{\text{n}}\sum_{i=1}^{n}(X_{i}-Y_{i}{)}^{\text{2}}} \end{array}$$ (6)

$$\begin{array}{c} MAE=\frac{\text{1}}{n}\sum_{i=1}^{n}\left|X_{i}-Y_{i}\right| \end{array}$$ (7)

$$\begin{array}{c} \text{ubRMSE}=\sqrt{\frac{\text{1}}{n}\sum_{i=1}^{n}{\left(X_{i}-{\overset{―}{X}}_{\text{i}}-(Y_{i}-{\overset{―}{Y}}_{i})\right)}^{\text{2}}} \end{array}$$ (8)

Where n represents the total number of samples;$X_{i}$ and$Y_{i}$ denote the  i-th observed and predicted soil moisture values, respectively; and ${\overset{―}{X}}_{i}$ and $\;{\overset{―}{Y}}_{\text{i}}$ signify the mean values of the observed and predicted soil moisture, respectively.

## Results

This section presents the experimental findings of the seasonally decoupled soil moisture retrieval. The following subsections detail the seasonal sensitivity of multi-source features, the performance comparison of the five machine learning models, and the physical mechanisms revealed through SHAP-based attribution analysis.

### Spatiotemporal distribution characteristics of precipitation in the study area

**Fig 3:**
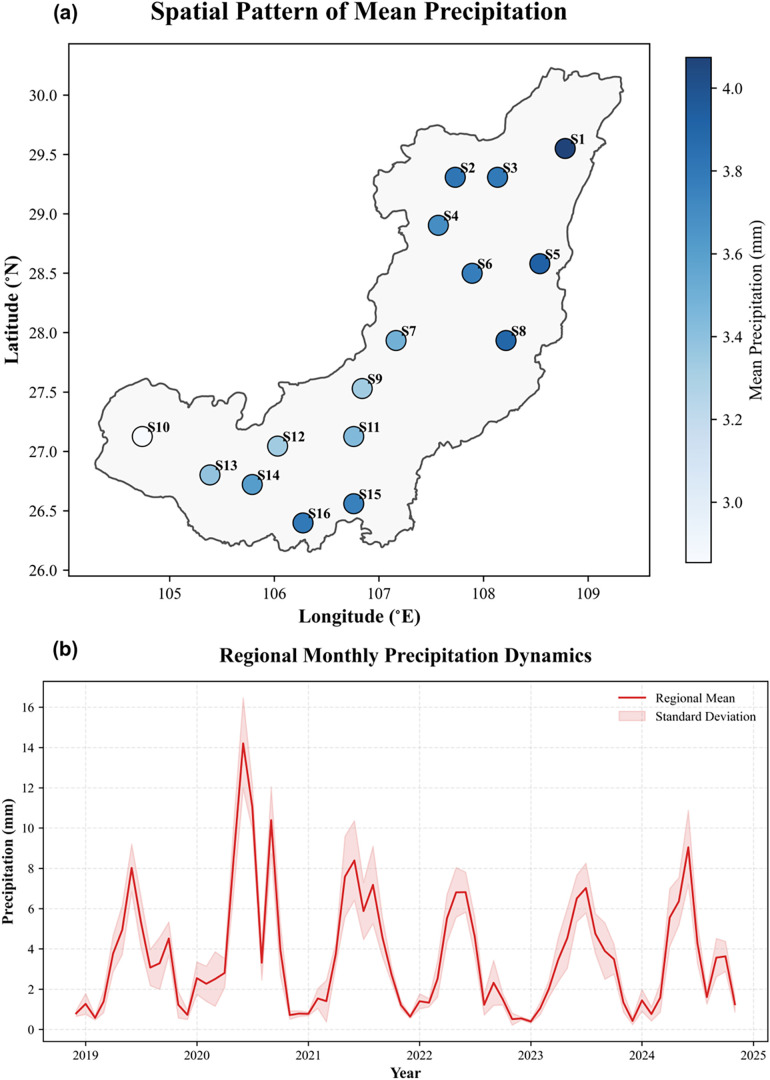
Spatiotemporal characteristics of precipitation in the Wujiang River Basin. **(a)** Spatial distribution of the multi-year average precipitation; **(b)** Dynamic variations of regional monthly precipitation from 2019 to 2024.

Based on the precipitation data analysis from 2019 to 2024 (Fig 3), the Wujiang River Basin exhibits pronounced spatial heterogeneity and intense temporal pulse fluctuations, which impose complex environmental constraints on soil moisture (SM) retrieval. Spatially, as illustrated in Fig 3(a), the multi-year average precipitation follows a decreasing gradient from south to north, with high-value areas primarily concentrated in the southern mountainous fringes. The distribution of stations S1–S16, covering an elevation gradient from the upper to the lower reaches, objectively records this significant spatial heterogeneity. Such disparities in precipitation baselines necessitate robust spatial generalization capabilities of the models to accurately capture SM characteristics across the highly fragmented Karst terrain. Temporally, Fig 3(b) reveals a highly uneven intra-annual distribution, with the summer season (May–September) contributing over 70% of the annual total, accompanied by substantial interannual non-linear fluctuations, while the winter remains under a prolonged low-moisture baseline.

The aforementioned spatiotemporal heterogeneity serves as a primary environmental driver for the seasonal fluctuations in model performance. In summer, frequent and intense precipitation pulses potentially induce a “signal decoupling” between surface thermal feedback signals and deep-layer moisture dynamics. This phenomenon likely exacerbates the non-linear complexity for models attempting to capture SM variations based solely on surface characteristics (e.g., LST and TVDI). In contrast, during the winter months, the extremely low moisture baseline combined with diminished vegetative physiological activity results in a marked decline in the sensitivity of dynamic remote sensing indicators (e.g., NDVI and moisture indices). Consequently, model predictions may become more susceptible to static geographic priors such as topography, potentially limiting the precision in capturing subtle local moisture fluctuations. These distinct seasonal variations in physical mechanisms provide a scientific rationale for developing the seasonally adaptive retrieval framework proposed in this study, offering a critical logical basis for the subsequent seasonally decoupled modeling.

### Importance selection of SMAP soil moisture and feature variables

**Fig 4:**
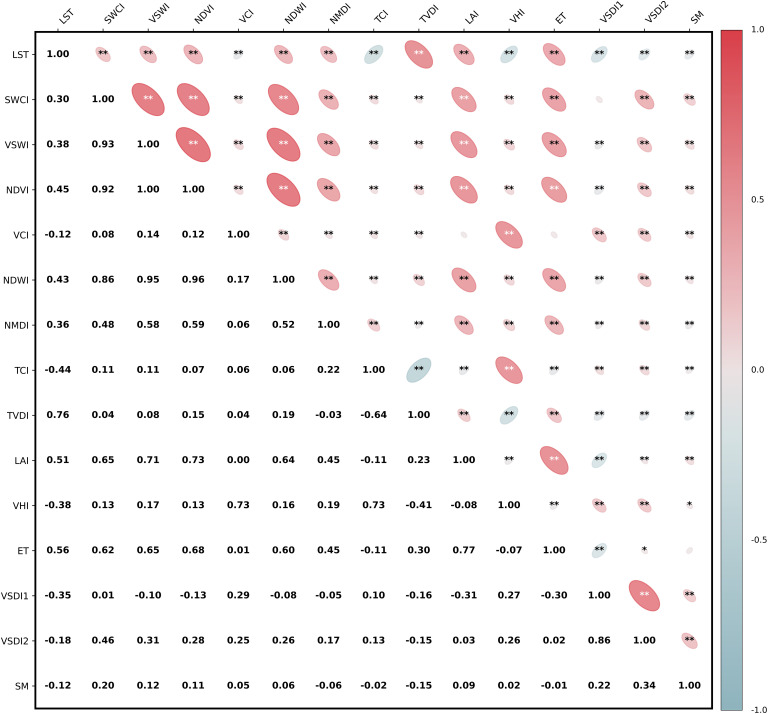
Correlation heatmap between remote sensing indicators and soil moisture in spring. ** and * indicate significance levels at 1% and 5%, respectively.

In this study, the linear correlations between soil moisture (SM) and 14 candidate remote sensing indicators across different seasons in the Wujiang River Basin were systematically evaluated using Pearson correlation analysis (Fig 4 and S1–S3 Figs). The results indicate that the predictive capacity of these remote sensing indicators exhibits significant seasonal variations. In spring (Fig 4), the Vegetation Surface Drought Index 2 (VSDI2,r=0.34) and the Surface Water Content Index (SWCI,r=0.20) demonstrated the strongest positive correlations, emerging as the dominant factors for this season. Conversely, the negative correlation of the Temperature Vegetation Dryness Index (TVDI,r=−0.15) highlights the surface thermal stress effects associated with rising temperatures. During summer (S1 Fig), the sensitivity of remote sensing features to SM was markedly weakened due to the stochastic interference of frequent precipitation and the saturation of vegetation signals; with the exception of Land Surface Temperature (LST,r=−0.24), which maintained a moderate negative correlation, the absolute correlation coefficients (|r|) for all other dynamic features remained below 0.25. In autumn (S2 Fig), the correlations showed a recovery, primarily driven by moisture indices VSDI1 (r=0.29) and VSDI2 (r=0.29), followed by the influence of LST (r=−0.24). In winter (S3 Fig), the surface environment appeared more constrained by geographical background factors, with TVDI (r=−0.21) and the Normalized Multi-band Drought Index (NMDI,r=0.22) providing the most effective indications of SM. Based on the identification of these seasonally sensitive features, this study established a differentiated input strategy comprising “three fundamental geographic features (Slope, Aspect, and DEM) plus six prioritized seasonal dynamic features.” This strategy provides a robust data foundation for constructing seasonal machine learning retrieval models supported by physical logic.

### Construction and comparison of soil moisture retrieval models

In this study, the SMAP L3 soil moisture product was utilized exclusively as the target variable (dependent variable) for model training to provide macroscopic moisture dynamics as supervision signals. Crucially, the original SMAP observations were strictly excluded from the input feature set (independent variables). Instead, the models were driven by 14 MODIS-derived dynamic indicators encompassing vegetation, temperature, moisture, and evapotranspiration attributes, supplemented by three fixed topographic features: elevation (DEM), slope, and aspect. This deliberate decoupling of inputs and targets aims to ensure that the framework independently learns the nonlinear associations between high-resolution land surface constraints and moisture signals, rather than performing a simple identity mapping of homologous data. In other words, the model is designed to learn statistical and physical patterns aligned with SMAP observations, while being entirely driven by independent, high-resolution environmental inputs.

To account for the pronounced phenological characteristics and climatic variations in the Wujiang River Basin, the 2019–2024 long-term sequence was partitioned into four periods based on meteorological standards. To maintain winter continuity, December data were reassigned to the subsequent “logical year” for unified modeling. Following rigorous quality control, a research database comprising 38,443 valid samples was established, with seasonal sample sizes of 9,663, 10,482, 9,343, and 8,955, respectively. For each seasonal dataset, a fixed random seed was employed to maintain a consistent 8:2 training-to-testing ratio, thereby ensuring the reproducibility and robustness of the experimental results. In compliance with data openness requirements, the minimal anonymized dataset supporting these findings has been provided as Supporting Information (see file S1 Data).

During the modeling process, a seasonally independent configuration strategy was adopted. Moving beyond traditional manual empirical settings, the Optuna framework was utilized in conjunction with 3-fold cross-validation to perform systematic synergistic optimization of hyperparameters for five machine learning algorithms. This workflow aimed to achieve an ideal balance between model complexity and generalization capability through objective parameter space searching, thereby effectively mitigating overfitting risks and ensuring the robustness of the retrieval framework across varying seasonal environments and hydrothermal fluctuations. Given that the CatBoost model demonstrated the optimal and most stable retrieval accuracy in subsequent validations, Table 2 details its optimized key hyperparameter combinations across different seasons, including: number of iterations (Iter), maximum tree depth (Depth), learning rate (LR), L2 leaf regularization coefficient (L2), and random strength (RS). These core parameters collectively determined the learning efficiency and noise resistance of the model in capturing the nonlinear characteristics of surface moisture. To maintain conciseness, the final parameter configurations of the other four comparative models (XGBoost, RF, SVM, and RBFNN) have been compiled in S1 Appendix.

**Table 2:** Optimized hyperparameter configurations for the machine learning models based on Optuna.

Season	Iterations (Iter)	Max Depth (Depth)	Learning Rate (LR)	L2 Leaf Reg (L2)	Random Strength (RS)
Spring	1908	6	0.076	25.73	2.52
Summer	1531	6	0.010	47.94	6.33
Autumn	1664	9	0.025	14.75	8.63
Winter	1673	6	0.015	27.56	1.00

The model performance was comprehensively evaluated using three standard metrics: the coefficient of determination ($R^{\text{2}}$), root mean square error (RMSE), and mean absolute error (MAE). Specifically, *R*² measures the model’s capability to explain the spatio-temporal variability of soil moisture; RMSE highlights the overall deviation between predicted and observed values with higher sensitivity to large errors; and MAE directly represents the average absolute magnitude of the retrieval errors. By statistically analyzing these metrics across both training and testing sets, the seasonal adaptability and generalization performance of the retrieval models in typical Karst basins were evaluated from three dimensions: variance explanation, bias magnitude, and average accuracy.

Table 3 summarizes the evaluation results of the five machine learning models under the seasonal modeling strategy. Overall, the models exhibited relatively favorable retrieval potential during spring and autumn ($R^{\text{2}}>\;0.40$), whereas their performance in summer and winter suggests room for further optimization. During the summer (June–August), the concentrated and intense precipitation within the Wujiang River Basin induced pronounced non-linear fluctuations in soil moisture. This phenomenon potentially led to a degree of “decoupling” or signal saturation between remote sensing features and deep soil moisture dynamics, resulting in test $R^{\text{2}}$ values typically ranging from 0.269 to 0.314. The retrieval challenges in winter may stem from the attenuation of land surface energy exchange under conditions of low temperature and weak solar radiation, which subsequently weakens the indicative capacity of thermal and optical features regarding moisture status. Furthermore, although the “logical year” approach for cross-year data maintained temporal continuity, the complex heterogeneity of the underlying surface posed a certain challenge to the generalization stability of models such as Random Forest and XGBoost.

**Table 3:** Seasonal comparison of retrieval accuracy among different machine learning models.

Model	Season	Train			Test		
		$R^{2}$	RMSE	MAE	$R^{2}$	RMSE	MAE
RBFNN	Spring	0.483	0.047	0.037	0.410	0.051	0.040
	Summer	0.441	0.050	0.038	0.314	0.053	0.041
	Autumn	0.531	0.052	0.039	0.445	0.056	0.043
	Winter	0.423	0.044	0.034	0.333	0.047	0.037
RF	Spring	0.598	0.041	0.032	0.467	0.048	0.038
	Summer	0.442	0.050	0.038	0.269	0.055	0.042
	Autumn	0.654	0.045	0.033	0.525	0.052	0.039
	Winter	0.439	0.043	0.034	0.286	0.048	0.038
SVM	Spring	0.463	0.048	0.037	0.406	0.051	0.04
	Summer	0.410	0.051	0.038	0.285	0.054	0.042
	Autumn	0.521	0.052	0.039	0.450	0.056	0.042
	Winter	0.320	0.048	0.037	0.279	0.049	0.038
CatBoost	Spring	0.663	0.038	0.030	0.537	0.045	0.035
	Summer	0.432	0.050	0.039	0.295	0.054	0.041
	Autumn	0.699	0.042	0.031	0.572	0.049	0.036
	Winter	0.453	0.043	0.033	0.302	0.048	0.038
XGBoost	Spring	0.604	0.041	0.032	0.504	0.046	0.037
	Summer	0.460	0.049	0.038	0.295	0.054	0.041
	Autumn	0.656	0.044	0.034	0.537	0.051	0.039
	Winter	0.458	0.042	0.034	0.305	0.048	0.038

In the comparison of model performance, the CatBoost model demonstrated encouraging adaptability in terms of cross-seasonal robustness and accuracy balance. Experimental data indicated that CatBoost achieved relatively high retrieval accuracy in spring ($R^{\text{2}}=\;0.537$) and autumn ($R^{\text{2}}=\;0.572$), exhibiting a more stable feature-capture capability compared to other evaluated algorithms. In contrast, while SVM performed reasonably well in autumn, its applicability in winter appeared somewhat limited; similarly, the accuracy of RBFNN was noticeably constrained during the summer months. In summary, benefiting from its symmetric tree structure and sophisticated parameter optimization strategy, the CatBoost model was able to relatively effectively characterize the complex surface hydrothermal coupling features across different phenological periods, serving as a promising reference algorithm for soil moisture retrieval within the framework of this study.

### Model generalization assessment based on leave-one-year-out (LOYO) cross-validation

Based on the aforementioned algorithmic comparisons, the CatBoost model demonstrated superior robustness. To further investigate its extrinsic extrapolation capability in the temporal dimension and mitigate potential over-fitting risks, a Leave-One-Year-Out (LOYO) strategy was employed for rigorous testing. Utilizing the 2019–2024 research sequence, an independent year was sequentially reserved as the validation set, with the Optuna framework integrated for dynamic hyperparameter optimization within each cycle. This approach seeks to preliminarily simulate the model's adaptive capacity to unseen meteorological backgrounds. The validation results (Table 4) suggest that CatBoost possesses promising generalization potential during the spring and autumn seasons, where land-surface hydrothermal coupling remains relatively stable. In the spring sequence, the mean $R^{\text{2}}$ reached 0.505, with annual fluctuations ranging from 0.454 to 0.545 and RMSE stabilizing between 0.042 and 0.046.

**Table 4:** Accuracy statistics of the CatBoost model using leave-one-year-out (LOYO) cross-validation.

Validation Year	Season	$R^{2}$	RMSE	MAE
2019	Spring	0.463	0.045	0.035
	Autumn	0.494	0.041	0.031
2020	Spring	0.545	0.042	0.033
	Autumn	0.492	0.047	0.037
2021	Spring	0.537	0.046	0.035
	Autumn	0.494	0.040	0.031
2022	Spring	0.544	0.043	0.036
	Autumn	0.491	0.045	0.034
2023	Spring	0.454	0.043	0.034
	Autumn	0.305	0.047	0.033
2024	Spring	0.487	0.044	0.037
	Autumn	0.486	0.043	0.032

In the autumn validation sequence, the $R^{\text{2}}$ performance remained relatively balanced at approximately 0.50, although the predictive robustness for 2023 indicates room for further improvement due to extreme inter-annual climatic variability. Such fluctuations in accuracy imply that by strengthening regularization constraints and parameter optimization, the model exhibits a certain smoothing capability when handling precipitation anomalies or temperature fluctuations, thereby effectively alleviating the significant accuracy oscillations observed in earlier experiments. The MAE averaged across the six-year autumn validation sequence remained stable at approximately 0.033. These findings preliminarily confirm the applicability of the seasonal retrieval framework integrating multi-source features within the Karst landforms of the Wujiang River Basin, potentially offering a methodological reference for long-term soil moisture monitoring in this region.

### Multi-source feature grouping ablation experiments

To further evaluate the specific contributions of different feature groups to the model's retrieval capability and to verify the rationality of the selected parameters, ablation experiments were conducted based on the CatBoost model, which exhibited the best overall performance. According to their physical significance, the input variables were categorized into six dimensions: Terrain (Dem, Slope, Aspect), Vegetation (NDVI, VCI, VHI, LAI), Moisture (NDWI, NMDI, VSDI1, VSDI2), Temperature (TCI, TVDI, LST), Composite Drought Indices (SWCI, VSWI), and Evapotranspiration (ET). Maintaining consistency with the original dataset partition (8:2) and the Optuna-based Bayesian hyperparameter optimization framework, each category of features was systematically excluded to perform feature re-selection and model re-training. The analysis primarily focused on the error variations in the test sets for spring and autumn, which previously demonstrated superior retrieval performance.

As summarized in Table 5, the exclusion of different feature groups led to varying degrees of decline in model fitting performance. Specifically, the removal of the Terrain feature group resulted in the most significant performance degradation; the $R^{\text{2}}$ for the autumn test set plummeted from a baseline of 0.604 to 0.478. This suggests that topographic factors play a crucial role in characterizing the complex spatial heterogeneity of soil moisture in Karst mountainous regions. Furthermore, when the Temperature or ET feature groups were removed, the autumn $R^{\text{2}}$ decreased to 0.504 and 0.525, respectively, indicating that land surface thermal states and water vapor flux indicators provide indispensable explanatory variables within the nonlinear surface hydrothermal relationships. Conversely, the performance attenuation observed upon removing Vegetation or Moisture indicators was relatively minor. This could potentially be attributed to a degree of information redundancy between the composite drought indices introduced in this study and the individual vegetation or moisture indices. The results of the ablation experiments quantitatively confirm that integrating multi-source remote sensing features can effectively capture the dynamic evolution of soil moisture, thereby enhancing the robustness and representational capacity of the model.

**Table 5:** Comparison of ablation experiment results for the CatBoost model via feature grouping during spring and autumn.

Ablation Group	Season	Train			Test		
		$R^{2}$	RMSE	MAE	$R^{2}$	RMSE	MAE
Composite	Spring	0.803	0.029	0.022	0.617	0.041	0.032
	Autumn	0.795	0.034	0.025	0.604	0.048	0.035
w/o Terrain	Spring	0.599	0.041	0.034	0.483	0.046	0.042
	Autumn	0.658	0.044	0.033	0.478	0.055	0.040
w/o Vegetation	Spring	0.786	0.030	0.023	0.597	0.042	0.033
	Autumn	0.795	0.034	0.025	0.601	0.047	0.035
w/o Temperature	Spring	0.740	0.033	0.026	0.567	0.043	0.034
	Autumn	0.691	0.042	0.031	0.504	0.054	0.041
w/o Moisture	Spring	0.782	0.030	0.024	0.598	0.042	0.033
	Autumn	0.777	0.036	0.026	0.585	0.049	0.036
w/o ET	Spring	0.755	0.032	0.025	0.573	0.043	0.034
	Autumn	0.729	0.039	0.029	0.525	0.052	0.039

Note: The row labeled “Composite” represents the comprehensive retrieval framework utilizing the complete feature set, serving as the benchmark for comparing other feature-removal scenarios.

### Interpretation of model decision mechanisms based on SHAP

To further investigate the decision-making logic of the CatBoost model for soil moisture (SM) retrieval in the Wujiang River Basin, this study employed the SHAP (SHapley Additive exPlanations) method for a comprehensive attribution analysis. By quantifying the contribution of each feature variable, the SHAP framework elucidates the internal mechanisms by which the model integrates multi-source remote sensing information. It is important to emphasize that SHAP analysis primarily clarifies the statistical dependencies within the model rather than establishing direct physical causality; its core value lies in evaluating the logical consistency between data-driven patterns and established hydro-geological principles.

**Fig 5:**
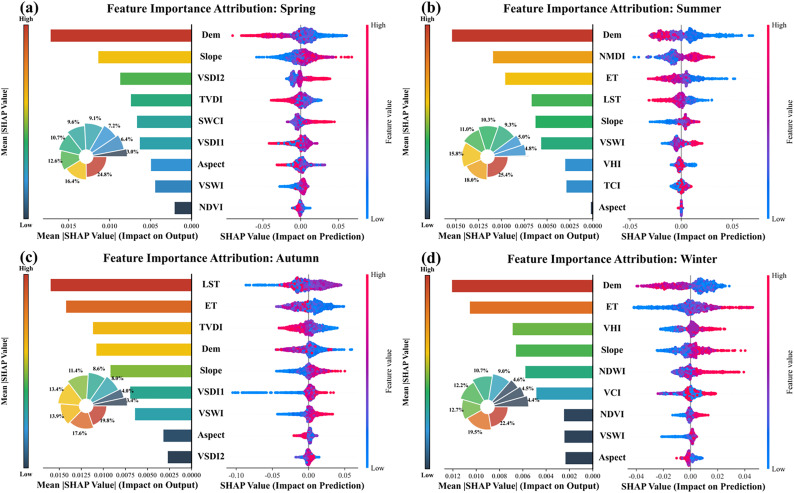
Seasonal analysis of feature contributions for soil moisture retrieval based on the CatBoost model in the Wujiang River Basin: (a) Spring; (b) Summer; (c) Autumn; (d) Winter.

As illustrated in Fig 5, the model's reliance on specific features exhibits a distinct seasonal adaptation, which appears to be closely associated with fluctuations in its predictive performance. Topographic factors (DEM, slope, and aspect) constitute a robust geographic baseline for the retrieval process, with the contribution of DEM remaining stable across all seasons (ranging from 13.4% to 25.4%). This sustained statistical sensitivity suggests a macro-control effect of the complex Karst micro-topography on water redistribution. During the spring and autumn seasons, characterized by higher retrieval accuracies, the model effectively synergizes static topographic backgrounds with dynamic moisture indices (e.g., SWCI and VSDI1). Such balanced integration of multi-source information likely underpins the enhanced predictive reliability during these periods.

In contrast, the retrieval task faces increased challenges during summer and winter, where a notable shift in the model's decision-making priority is observed. In summer, the contribution of Land Surface Temperature (LST) and TVDI increases substantially, reflecting a high dependence on thermal characteristic signals under high-temperature conditions. However, frequent flash rain events in summer may lead to a “signal decoupling” between surface thermal indicators and deep-layer soil moisture evolution. This non-linear fluctuation in hydrothermal relationships potentially explains the relative attenuation of model accuracy during the summer months. Furthermore, the beeswarm plots (Fig 5, right) reveal the directional influence of these drivers: in summer and autumn, high values (red points) of LST and TVDI are densely clustered in the negative SHAP zone. This negative “dry-warm” correlation logic is highly consistent with known hydro-physical patterns in Karst regions. The positive predictive gain provided by moisture indices, along with the non-linear clustering of micro-topographic features around the zero axis, suggests that the model has captured the subtle regulatory effects of heterogeneous environments on local humidity. In summary, the SHAP attribution analysis outlines a decision-making framework that aligns statistical evidence with physical plausibility, demonstrating the capacity of the CatBoost model to identify statistical patterns that are consistent with known seasonal hydrological behaviors through adaptive feature weighting.

### Station-based model validation and performance evaluation

**Fig 6:**
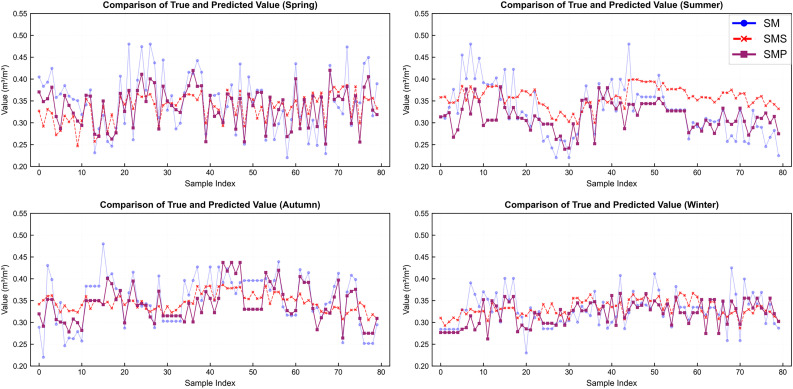
Comparison of observed SM, original SMAP, and CatBoost predicted soil moisture across four seasons (2019–2020).

To evaluate the applicability of the retrieval models at the station scale, daily observational data from 16 ground stations during 2019–2020 were collected and aggregated into benchmark values (SMS) using the median method to match the spatiotemporal scale of the remote sensing products. Taking these as a reference, the statistical performances of the original SMAP product (SM) and the machine learning-based predictions (SMP) were systematically compared using metrics including ubRMSE, RMSE, and MAE (Table 6). Within this framework, SM-SMS and SMP-SMS reflect the respective deviations of the original satellite products and the model retrieval results from the ground-based measurements. Furthermore, Fig 6 presents the time-series comparison curves of SM, SMS, and SMP during the corresponding periods, aiming to qualitatively evaluate the capability of different models in capturing the dynamic evolution of soil moisture through the fluctuation synergy among the three curves.

**Table 6:** Station-scale statistical performance of the five machine learning models (SMP) versus the original SMAP product (SM).

Evaluation	Season	SM-SMS	SMP-SMS				
			RBFNN	RF	SVM	CatBoost	XGBoost
ubRMSE	Spring	0.0632	0.0480	0.0414	0.0474	0.0482	0.0436
	Summer	0.0530	0.0333	0.0272	0.0318	0.0312	0.0317
	Autumn	0.0531	0.0447	0.0414	0.0463	0.0476	0.0421
	Winter	0.0386	0.0379	0.0262	0.0294	0.0307	0.0269
RMSE	Spring	0.0636	0.0481	0.0414	0.0474	0.0482	0.0437
	Summer	0.0606	0.0572	0.0588	0.0570	0.0599	0.0589
	Autumn	0.0533	0.0504	0.0497	0.0521	0.0520	0.0490
	Winter	0.0391	0.0421	0.0342	0.0355	0.0375	0.0350
MAE	Spring	0.0517	0.0397	0.0342	0.0392	0.0403	0.0359
	Summer	0.0518	0.0513	0.0540	0.0517	0.0546	0.0532
	Autumn	0.0413	0.0412	0.0416	0.0412	0.0429	0.0411
	Winter	0.0315	0.0337	0.0281	0.0298	0.0306	0.0286

The statistical results in Table 6 indicate that, compared with the original SMAP products, the predictive values generated by the five machine learning models exhibit varying degrees of improvement across all statistical metrics when validated against in-situ observations. Notably, while the SMAP product was employed as the macro-scale supervision target for model training, the original SMAP observational data were strictly excluded from the input features. This decoupled design between input features and the training target suggests that the integration of high-resolution auxiliary variables may effectively capture the surface heterogeneity within the original 9 km pixels that is typically smoothed out. Regarding temporal dynamic evolution, taking the CatBoost model as a primary example (Fig 6)—which demonstrated relatively favorable performance—the predicted (SMP) curves show a consistent trend with the SMS (results for the other four models are provided in S4–S7 Figs). Particularly during the spring and autumn seasons, when the hydrothermal coupling remains relatively stable, the fluctuation phases of both datasets show a high degree of consistency. These preliminary findings suggest that the modeling framework developed in this study, by integrating multi-source underlying surface characteristics, has the potential to capture the seasonal dynamic evolution of soil moisture.

## Discussion

This section interprets the experimental results within the context of Karst eco-hydrology and discusses the driving mechanisms identified through model attribution. The following subsections analyze the seasonal shifting of key environmental drivers, evaluate the advantages of the decoupled modeling framework, and acknowledge the potential limitations and future research directions of this study.

### Seasonal correlation and mechanisms of feature variables

The sensitivity of remote sensing indicators to soil moisture (SM) is profoundly regulated by land-surface water-thermal coupling mechanisms. Pearson correlation analysis reveals that the sensitivity of remote sensing factors exhibits significant seasonal differentiation. The higher sensitivity observed during spring and autumn (with |r| values for key indicators reaching 0.34 and 0.29, respectively, p<0.01) suggests that optical and thermal infrared indices can reliably capture surface water-thermal states during the early stages of moisture deficit and late stages of vegetation growth. In contrast, a pronounced “signal decoupling” phenomenon occurs during summer, where the maximum |r| among all indicators drops to only 0.24 (for LST), with most indicators exhibiting very weak correlations (mean|r|=0.116). This may be attributed, on one hand, to the susceptibility of optical signals to saturation under high temperature and humidity conditions; on the other hand, it stems from non-linear moisture fluctuations triggered by episodic heavy precipitation (Fig 3), leading to a temporal mismatch between instantaneous remote sensing signals and ground truth moisture states. Quantitatively, the lower standard deviation of correlation coefficients in summer (SD=0.050) compared to spring (SD=0.092) reflects a consistent and widespread decline in feature sensitivity across almost all spectral dimensions. Furthermore, the persistent negative correlation of LST in autumn (r=−0.24,p<0.01) confirms the effectiveness of thermal response as an indicator of moisture stress. The relatively weak correlations of NDVI and LAI across most seasons (|r|≤0.17) reveal the lagged physiological response of vegetation in complex mountainous environments [63]. Such significant seasonal variability underscores the necessity of employing machine learning algorithms to integrate these multidimensional “weak signal” features.

### Multi-factor driving mechanisms of soil moisture

The soil moisture (SM) in the Wujiang River Basin is synergistically regulated by non-linear interactions among meteorological forcing, topographic redistribution, and land-surface water-thermal exchange processes. As the core variable driving moisture dynamics, precipitation exhibits significant spatiotemporal heterogeneity, which establishes the fundamental pattern of SM within the basin (Fig 3). During summer, intensive and high-frequency precipitation recharge causes the variation rate of surface SM to frequently exceed the capture threshold of MODIS indicators, making it difficult to effectively characterize the surface water-thermal synergistic mechanisms through quasi-static parameters. In contrast, the water-thermal dynamics during spring and autumn tend to be more moderate, allowing the model to more accurately extract the physical correlations between LST and vegetation-moisture indices. However, the characterization capability is somewhat constrained during winter due to vegetation dormancy and the diminished sensitivity of surface thermal response to moisture fluctuations. Furthermore, the typical Karst mountainous topography intensifies the spatial redistribution of water, exacerbating signal heterogeneity within pixels. By integrating topographic features such as elevation (DEM), slope, and aspect, this study effectively compensates for the limited explanatory power of individual indices over complex underlying surfaces, revealing the continuous regulatory role of geological conditions on moisture dynamics. This analysis of driving mechanisms suggests that future research could explicitly incorporate higher-precision geological parameters into the existing feature fusion framework to deepen the characterization of moisture movement mechanisms in Karst mountainous areas, thereby enhancing the robustness of monitoring in complex habitats [64].

### Assessment of seasonal adaptability in machine learning models

In the complex habitats of the Wujiang River Basin, characterized by drastic hydrothermal dynamics and highly fragmented terrain, machine learning techniques demonstrate more robust applicability than traditional models by capturing deep nonlinear patterns among variables. A comparative analysis of five algorithms indicates that the CatBoost model achieves a relatively ideal balance of accuracy within the “seasonally decoupled” framework. During the spring and autumn seasons, when hydrothermal conditions are moderate and phenological responses are sensitive, the model’s test set $R^{\text{2}}$ reached 0.537 and 0.572, respectively, suggesting a enhanced capacity for capturing spatial heterogeneity compared to baseline models. Furthermore, Leave-One-Year-Out (LOYO) cross-validation, conducted through independent annual testing from 2019 to 2024, provides preliminary evidence of the model’s potential for temporal extrapolation in response to interannual climatic fluctuations. Although the model's explanatory power declines during extreme periods—impacted by signal “decoupling” in summer and reduced thermal detection sensitivity in winter—it maintains fundamental retrieval robustness by integrating topographic and hydrothermal features through the gradient boosting mechanism. SHAP attribution analysis further confirms that the decision logic of this framework is highly consistent with land-surface physical processes: during spring and winter, the model prioritizes static geographic background constraints, reflecting the dominant role of topographic redistribution during periods of low phenological activity; in contrast, during summer and autumn, the model more effectively captures dynamic hydrothermal signals. This logical evolution from “topography-driven” to “hydrothermal-driven” mechanisms helps mitigate the spatial representation errors of coarse-scale products in complex terrains, thereby enhancing the credibility of data-driven models for applications in Karst mountainous regions.

### Limitations and future outlook

There remains scope for further enhancement in this study regarding data sources, spatiotemporal resolution, and model validation. The model relies heavily on optical remote sensing features; however, frequent precipitation and persistent cloud cover during the summer in the Wujiang River Basin degrade the quality and availability of optical imagery, directly limiting retrieval accuracy during the wet season. Furthermore, the scale mismatch between 9 km SMAP products and MODIS factors may introduce mixed-pixel errors, and the 8-day revisit cycle struggles to capture instantaneous fluctuations in soil moisture. In terms of validation, the spatial representativeness gap between point-scale measurements and pixel-scale retrievals persists due to the limited distribution of ground stations.

To address these challenges, future research could integrate multi-band and multi-angle microwave remote sensing (e.g., Sentinel-1 SAR) to overcome cloud and rain observation bottlenecks. Focusing on the spring and autumn seasons, characterized by relatively stable hydrothermal dynamics, may provide a more favorable window for revealing the underlying mechanisms of soil moisture retrieval in Karst mountainous areas. Given the highly fragmented and complex habitats, the model’s capacity to extract deep non-linear features warrants further reinforcement. Advanced feature enhancement strategies, such as utilizing contrastive learning to construct more discriminative semantic representations [65], could be adopted to deepen feature extraction over complex underlying surfaces. Additionally, to address the robustness bottlenecks caused by summer signal decoupling, it is advisable to explore modeling pathways that integrate complex logical constraints. This could involve introducing reinforcement learning and adaptive loss functions to dynamically optimize learning strategies [66], thereby constructing a deep learning framework that balances physical constraints with data-driven patterns. Such deep coupling of multi-source data and frontier algorithms will help mitigate pixel-scale representation errors and systematically enhance the accuracy and reliability of soil moisture monitoring in Karst mountainous regions.

## Conclusion

This study developed and evaluated a seasonal machine learning-based retrieval framework integrating multi-source MODIS features to address the limitations of satellite-derived soil moisture (SM) products in complex Karst terrains, using the Wujiang River Basin as a representative study area. The primary conclusions are as follows:

(1) The seasonal sensitivity variations in the coupling between land-surface features and SM were elucidated. The findings indicate that the explanatory power of remote sensing indicators is modulated by regional hydrothermal dynamics, exhibiting pronounced seasonal differentiation. Surface thermal and vegetation features show high sensitivity during spring and autumn, whereas a notable “signal decoupling” occurs in summer due to non-linear perturbations from intensive precipitation. This provides a physical rationale for developing seasonally decoupled retrieval models.
(2) The applicability of the CatBoost algorithm for representing non-linear associations over complex underlying surfaces was verified. Compared to other machine learning methods, the CatBoost model demonstrates a relatively ideal balance of accuracy and robustness across seasons. Despite performance fluctuations during summer and winter due to extreme habitats and transient rainfall, the model effectively integrates topographic and thermal features through its gradient boosting mechanism, identifying a statistical shift in importance from ‘topography-driven’ to ‘hydrothermal-driven’ patterns.
(3) The effectiveness of the seasonal retrieval framework in mitigating pixel-scale representation errors was confirmed. Independent validation at the station scale shows that the retrieved results exhibit higher consistency with ground observations across all seasons compared to the original SMAP products. By integrating high-resolution auxiliary data, the framework partially compensates for the loss of surface heterogeneity within original coarse-scale pixels. Future research should further explore deep learning strategies that incorporate physical constraints to continuously enhance the stability and generalization potential of SM monitoring in complex environments.

## Supporting information

S1 Fig Correlation heatmap between remote sensing indicators and soil moisture in Summer. ** and * indicate significance levels at 1% and 5%, respectively.
(PDF)

S2 Fig Correlation heatmap between remote sensing indicators and soil moisture in Autumn. ** and * indicate significance levels at 1% and 5%, respectively.
(PDF)

S3 Fig Correlation heatmap between remote sensing indicators and soil moisture in Winter. ** and * indicate significance levels at 1% and 5%, respectively.
(PDF)

S4 Fig Comparison of observed SM, original SMAP, and predicted SM by RBFNN model across four seasons (2019–2020). (PDF)

S5 Fig Comparison of observed SM, original SMAP, and predicted SM by RF model across four seasons (2019–2020). (PDF)

S6 Fig Comparison of observed SM, original SMAP, and predicted SM by SVM model across four seasons (2019–2020). (PDF)

S7 Fig Comparison of observed SM, original SMAP, and predicted SM by XGBoost model across four seasons (2019–2020). (PDF)

S1 Data Anonymized minimal dataset for soil moisture retrieval analysis. (CSV)

S1 Appendix Seasonal optimal hyperparameters for comparative machine learning models. (DOCX)

## References

[1] Li N, Skaggs TH, Ellegaard P, Bernal A, Scudiero E. Relationships among soil moisture at various depths under diverse climate, land cover and soil texture. Sci Total Environ. 2024;947:174583. doi: 10.1016/j.scitotenv.2024.174583 38981543

[2] Osenga EC, Cohen AL, Clow DW, Deems JS, Jablonski JJ, Goble PD. Bioclimatic and soil moisture monitoring across elevation in a mountain watershed: The Roaring Fork catchment, Colorado, USA. Water Resources Research. 2019;55(11):9355–72. doi: 10.1029/2019WR025064

[3] Zhu B, Xie X, Meng S, Lu C, Yao Y. Sensitivity of soil moisture to precipitation and temperature over China: Present state and future projection. Sci Total Environ. 2020;705:135774. doi: 10.1016/j.scitotenv.2019.135774 31972934

[4] Wu Z, Cui N, Zhang W, Gong D, Liu C, Liu Q, et al. Inversion of large-scale citrus soil moisture using multi-temporal Sentinel-1 and Landsat-8 data. Agricultural Water Management. 2024;294:108718. doi: 10.1016/j.agwat.2024.108718

[5] Jia T, Shamseldin AY, Liu T, Bao Y, Wang Z, Duan L. Soil moisture inversion method for semi-arid regions using multi-temporal Sentinel-1 and Sentinel-2 data. J Hydrol. 2025;661:133603. doi: 10.1016/j.jhydrol.2025.133603

[6] Zheng Y, Fattahi H. Modeling, prediction, and retrieval of surface soil moisture from InSAR closure phase. Remote Sens Environ. 2026;333:115104. doi: 10.1016/j.rse.2025.115104

[7] Rahmati M, Balenzano A, Bechtold M, Brocca L, Fluhrer A, Jagdhuber T, et al. Soil moisture retrieval from Sentinel-1: Lessons learned after more than a decade in orbit. Remote Sensing of Environment. 2026;333:115146. doi: 10.1016/j.rse.2025.115146

[8] Shen Z, He Q, Yang C, Cheng Z. Soil moisture retrieval under different land cover conditions based on Sentinel-1 SAR. Remote Sensing of Environment. 2026;333:115147. doi: 10.1016/j.rse.2025.115147

[9] Balenzano A, Mattia F, Satalino G, Lovergine FP, Palmisano D, Peng J. Sentinel-1 soil moisture at 1 km resolution: a validation study. Remote Sens Environ. 2021;263:112554. doi: 10.1016/j.rse.2021.112554

[10] Peng J, Albergel C, Balenzano A, Brocca L, Cartus O, Cosh MH, et al. A roadmap for high-resolution satellite soil moisture applications – confronting product characteristics with user requirements. Remote Sensing of Environment. 2021;252:112162. doi: 10.1016/j.rse.2020.112162

[11] Kganyago M, Adjorlolo C, Mhangara P, Tsoeleng L. Optical remote sensing of crop biophysical and biochemical parameters: An overview of advances in sensor technologies and machine learning algorithms for precision agriculture. Comput Electron Agric. 2024;218:108730. doi: 10.1016/j.compag.2024.108730

[12] Zhu Z, Eylander J, Lakshmi V. A global framework for subsurface soil moisture estimation: Coupling fractal Richards equation with Bayesian optimization. Remote Sensing of Environment. 2026;336:115318. doi: 10.1016/j.rse.2026.115318

[13] Xie F, Fan H. Deriving drought indices from MODIS vegetation indices (NDVI/EVI) and Land Surface Temperature (LST): Is data reconstruction necessary? Int J Appl Earth Obs Geoinf. 2021;101:102352. doi: 10.1016/j.jag.2021.102352

[14] Wang J, Bi J, Lu L, Yao Y. Vegetation supply water index based on MODIS data analysis of the in Yunnan in spring of 2012. In: 2014 Third International Conference on Agro-Geoinformatics, 2014. 1–7. doi: 10.1109/Agro-Geoinformatics.2014.6910673

[15] Li Z, Mu Z, Qiu X, Liu J. Changes in future drought characteristics in the Ili River Basin, China, using the new comprehensive standardized drought index. Ecol Indic. 2025;173:113412. doi: 10.1016/j.ecolind.2025.113412

[16] Rendana M, Idris WMR, Alia F, Rahim SE, Yamin M, Izzudin M. Relationship between drought and soil erosion based on the normalized differential water index (NDWI) and revised universal soil loss equation (RUSLE) model. Reg Sustain. 2024;5(4):100183. doi: 10.1016/j.regsus.2024.100183

[17] Wang L, Qu JJ. NMDI: A normalized multi-band drought index for monitoring soil and vegetation moisture with satellite remote sensing. Geophysical Research Letters. 2007;34:L20405. doi: 10.1029/2007GL031021

[18] Li H, Kaufmann H, Xu G. Modeling spatio-temporal drought events based on multi-temporal, multi-source remote sensing data calibrated by soil humidity. Chin Geogr Sci. 2022;32(1):127–41. doi: 10.1007/s11769-021-1250-4

[19] Huang D, Ma T, Liu J, Zhang J. Agricultural drought monitoring using modified TVDI and dynamic drought thresholds in the upper and middle Huai River Basin, China. J Hydrol Reg Stud. 2025;57:102069. doi: 10.1016/j.ejrh.2024.102069

[20] Zeng J, Zhou T, Qu Y, Bento VA, Qi J, Xu Y, et al. An improved global vegetation health index dataset in detecting vegetation drought. Sci Data. 2023;10(1):338. doi: 10.1038/s41597-023-02255-3 37258520 PMC10232453

[21] Sun S, Bi Z, Mu M, Liu Y, Zhang Y, Li J. Quantifying impacts of vegetation greenness change on drought over global vegetation zones. Geophys Res Lett. 2025;52:e2024GL111634. doi: 10.1029/2024GL111634

[22] Zhou Z, Wang P, Li L, Fu Q, Ding Y, Chen P, et al. Recent development on drought propagation: A comprehensive review. J Hydrol. 2024;645:132196. doi: 10.1016/j.jhydrol.2024.132196

[23] Jahangir MH, Arast M. Estimation of surface soil moisture based on improved multi-index models and surface energy balance system. Nat Resour Res. 2021;30:789–804. doi: 10.1007/s11053-020-09728-x

[24] Bhagwat RU, Uma Shankar B. A novel multilabel classification of remote sensing images using XGBoost. In: 2019 IEEE 5th International Conference for Convergence in Technology (I2CT), 2019. 1–5. doi: 10.1109/i2ct45611.2019.9033768

[25] Samat A, Li E, Du P, Liu S, Miao Z, Zhang W. CatBoost for RS Image Classification With Pseudo Label Support From Neighbor Patches-Based Clustering. IEEE Geosci Remote Sensing Lett. 2022;19:1–5. doi: 10.1109/lgrs.2020.3038771

[26] Kandasamy L, Mahendran A, Sangaraju SHV, Mathur P, Faldu SV, Mazzara M. Enhanced remote sensing and deep learning aided water quality detection in the Ganges River, India supporting monitoring of aquatic environments. Results in Engineering. 2025;25:103604. doi: 10.1016/j.rineng.2024.103604

[27] Lary DJ, Alavi AH, Gandomi AH, Walker AL. Machine learning in geosciences and remote sensing. Geosci Front. 2016;7(1):3–10. doi: 10.1016/j.gsf.2015.07.003

[28] Tang Z, Zhang W, Xiang Y, Liu X, Wang X, Shi H, et al. Monitoring of Soil Moisture Content of Winter Oilseed Rape (Brassica napus L.) Based on Hyperspectral and Machine Learning Models. J Soil Sci Plant Nutr. 2024;24(1):1250–60. doi: 10.1007/s42729-024-01626-y

[29] Nguyen TT, Ngo HH, Guo W, Chang SW, Nguyen DD, Nguyen CT, et al. A low-cost approach for soil moisture prediction using multi-sensor data and machine learning algorithm. Sci Total Environ. 2022;833:155066. doi: 10.1016/j.scitotenv.2022.155066 35398433

[30] Wang X, Liu H, Sun Z, Han X. Soil moisture inversion based on multiple drought indices and RBFNN: A case study of northern Hebei Province. Heliyon. 2024;10(17):e37426. doi: 10.1016/j.heliyon.2024.e37426 39296096 PMC11409120

[31] Ettalbi M, Baghdadi N, Garambois P-A, Bazzi H, Ferreira E, Zribi M. Soil Moisture Retrieval in Bare Agricultural Areas Using Sentinel-1 Images. Remote Sensing. 2023;15(14):3502. doi: 10.3390/rs15143502

[32] Shi J, Yang H, Hou X, Zhang H, Tang G, Zhao H, et al. Coupling SAR and optical remote sensing data for soil moisture retrieval over dense vegetation covered areas. PLoS One. 2025;20(1):e0315971. doi: 10.1371/journal.pone.0315971 39820018 PMC11737747

[33] Yang Q, Chen J, Yang G, Xie H, Li M, Sun J. Dynamic evolution of rocky desertification and vegetation restoration and analysis of driving forces in Southwest Karst Region from 2000 to 2020. PLoS One. 2025;20(11):e0332644. doi: 10.1371/journal.pone.0332644 41237123 PMC12617925

[34] Ahmed M, Else B, Eklundh L, Ardö J, Seaquist J. Dynamic response of NDVI to soil moisture variations during different hydrological regimes in the Sahel region. International Journal of Remote Sensing. 2017;38(19):5408–29. doi: 10.1080/01431161.2017.1339920

[35] Zhang H, Chen H, Sun R, Yu W, Zou C, Shen S. The application of unified surface water capacity method in drought remote sensing monitoring. In: Proc SPIE, 2009. 74721M. doi: 10.1117/12.829735

[36] Pathak AA, Dodamani BM. Application of remotely sensed NDVI and soil moisture to monitor long-term agricultural drought. In: Proc SPIE, 2019. 111490P. doi: 10.1117/12.2532852

[37] Casamitjana M, Torres-Madroñero MC, Bernal-Riobo J, Varga D. Soil moisture analysis by means of multispectral images according to land use and spatial resolution on andosols in the Colombian Andes. Applied Sciences. 2020;10(16):5540. doi: 10.3390/app10165540

[38] Zhang H, Chen HL, Shen S. The application of Normalized Multi-band Drought Index (NMDI) method in cropland drought monitoring. In: Proc SPIE, 2009. 74721P. doi: 10.1117/12.830557

[39] Kukunuri ANJ, Murugan D, Singh D. Variance based fusion of VCI and TCI for efficient classification of agriculture drought using MODIS data. Geocarto International. 2020;37(10):2871–92. doi: 10.1080/10106049.2020.1837256

[40] Sun H, Gao JH, Yan TT, Hu KK, Xu ZH, Wang YJ. Remote sensing of vegetation drought: Research progress. Natl Remote Sens Bull. 2024;28(6):1395–411. doi: 10.11834/jrs.20243374

[41] Li Y, Wang X, Wang F, Feng K, Li H, Han Y, et al. Temporal and Spatial Characteristics of Agricultural Drought Based on the TVDI in Henan Province, China. Water. 2024;16(7):1010. doi: 10.3390/w16071010

[42] Patil PP, Jagtap MP, Khatri N, Madan H, Vadduri AA, Patodia T. Exploration and advancement of NDDI leveraging NDVI and NDWI in Indian semi-arid regions: A remote sensing-based study. Case Studies in Chemical and Environmental Engineering. 2024;9:100573. doi: 10.1016/j.cscee.2023.100573

[43] Kogan FN. Droughts of the late 1980s in the United States as derived from NOAA polar-orbiting satellite data. Bull Am Meteorol Soc. 1995;76(5):655–68. doi: 10.1175/1520-0477(1995)076<0655:DOTLIT>2.0.CO;2

[44] Chen Y, Xie L, Liu X, Qi Y, Ji X. Identification of High-Quality Vegetation Areas in Hubei Province Based on an Optimized Vegetation Health Index. Forests. 2024;15(9):1576. doi: 10.3390/f15091576

[45] Kallel A, Wang Y, Hedman J, Gastellu-Etchegorry JP. Canopy BRDF differentiation on LAI based on Monte Carlo Ray Tracing. Remote Sens Environ. 2025;327:114785. doi: 10.1016/j.rse.2025.114785

[46] Gao B. NDWI—A normalized difference water index for remote sensing of vegetation liquid water from space. Remote Sensing of Environment. 1996;58(3):257–66. doi: 10.1016/s0034-4257(96)00067-3

[47] Zhang N, Hong Y, Qin Q, Liu L. VSDI: a visible and shortwave infrared drought index for monitoring soil and vegetation moisture based on optical remote sensing. International Journal of Remote Sensing. 2013;34(13):4585–609. doi: 10.1080/01431161.2013.779046

[48] Kogan FN. Application of vegetation index and brightness temperature for drought detection. Advances in Space Research. 1995;15(11):91–100. doi: 10.1016/0273-1177(95)00079-t11539265

[49] Sandholt I, Rasmussen K, Andersen J. A simple interpretation of the surface temperature/vegetation index space for assessment of surface moisture status. Remote Sensing of Environment. 2002;79(2–3):213–24. doi: 10.1016/s0034-4257(01)00274-7

[50] Qin J, Wang D, Chang G, Li X. Correcting angular effects on MODIS LST in urban areas using an enhanced time-evolving parametric geometric model. Remote Sensing of Environment. 2026;332:115103. doi: 10.1016/j.rse.2025.115103

[51] Hong Z, Zhang W, Yu C, Zhang D, Li L, Meng L. SWCTI: Surface Water Content Temperature Index for Assessment of Surface Soil Moisture Status. Sensors (Basel). 2018;18(9):2875. doi: 10.3390/s18092875 30200308 PMC6164290

[52] Trisasongko BH, Panuju DR, Shiddiq D, Iman LOS, Sholihah RI, Kusdaryanto S. Constraints of VSWI in the Estimation of Drought Extent Using Landsat Data: A Case of Tuban, Indonesia. Procedia Environmental Sciences. 2015;24:25–8. doi: 10.1016/j.proenv.2015.03.004

[53] Joiner J, Yoshida Y, Anderson M, Holmes T, Hain C, Reichle R. Global relationships among traditional reflectance vegetation indices (NDVI and NDII), evapotranspiration (ET), and soil moisture variability on weekly timescales. Remote Sens Environ. 2018;219:339–52. doi: 10.1016/j.rse.2018.10.020 31217640 PMC6582971

[54] Dufera AG, Liu T, Xu J. Regression models of Pearson correlation coefficient. Statistical Theory and Related Fields. 2023;7(2):97–106. doi: 10.1080/24754269.2023.2164970

[55] Broomhead DS, Lowe D. Multivariable functional interpolation and adaptive networks. Complex Syst. 1988;2:321–55.

[56] Breiman L. Random Forests. Machine Learning. 2001;45(1):5–32. doi: 10.1023/a:1010933404324

[57] Chen T, Guestrin C. XGBoost: A scalable tree boosting system. In: Proceedings of the 22nd ACM SIGKDD International Conference on Knowledge Discovery and Data Mining, 2016. 785–94. doi: 10.1145/2939672.2939785

[58] Prokhorenkova L, Gusev G, Vorobev A, Dorogush AV, Gulin A. CatBoost: unbiased boosting with categorical features. Adv Neural Inf Process Syst. 2018;31:6638–48.

[59] Cortes C, Vapnik V. Support-vector networks. Mach Learn. 1995;20:273–97. doi: 10.1007/BF00994018

[60] Majumdar P. Plant Leaf Disease Detection Using XGBoost with OPTUNA Hyperparameter Optimization. SN COMPUT SCI. 2025;6(7). doi: 10.1007/s42979-025-04434-y

[61] Lundberg SM, Lee SI. A unified approach to interpreting model predictions. Adv Neural Inf Process Syst. 2017;30:4765–74.

[62] Raza Abbas S, Abbas Z, Zahir A, Ur Rehman M, Won Lee S. Phylogenomics to structure: evolutionary and clinical signals in the TP53 DNA-binding core through LOOCV-validated ensemble learning. Brief Bioinform. 2026;27(1):bbag087. doi: 10.1093/bib/bbag087 41744228 PMC12936793

[63] Zhang J, Hao FH, Wu ZF, Li MW, Zhang X, Fu YS. Response of vegetation phenology to extreme climate events and its mechanism: a review. Acta Geogr Sin. 2023;78(9):2125–44. doi: 10.11821/dlxb202309001

[64] Zhang R, Lv J, Yang Y, Wang T, Liu G. Analysis of the impact of terrain factors and data fusion methods on uncertainty in intelligent landslide detection. Landslides. 2024;21(8):1849–64. doi: 10.1007/s10346-024-02260-6

[65] Wang J, Wang B, Zhou S, Cao B, Li W, Zheng P. DNACSE: Enhancing Genomic LLMs with Contrastive Learning for DNA Barcode Identification. J Chem Inf Model. 2026;66(2):976–93. doi: 10.1021/acs.jcim.5c02747 41528854

[66] Liu X, Zheng Y, Li X, Wang B, Zhou S, Cao B, et al. An end-to-end DNA storage coding method based on a low-complexity multiple biological constraints loss and RL-inspired differentiable solver. Expert Systems with Applications. 2026;315:131726. doi: 10.1016/j.eswa.2026.131726

