# Supplementary figures and images for: Machine learning-based seasonal SMAP soil moisture retrieval integrating MODIS drought indices: A case study of the Wujiang River Basin
Source: PLoS One. 2026 Jun 22;21(6):e0351643. doi: 10.1371/journal.pone.0351643 (PMC13286200; doi:10.1371/journal.pone.0351643)

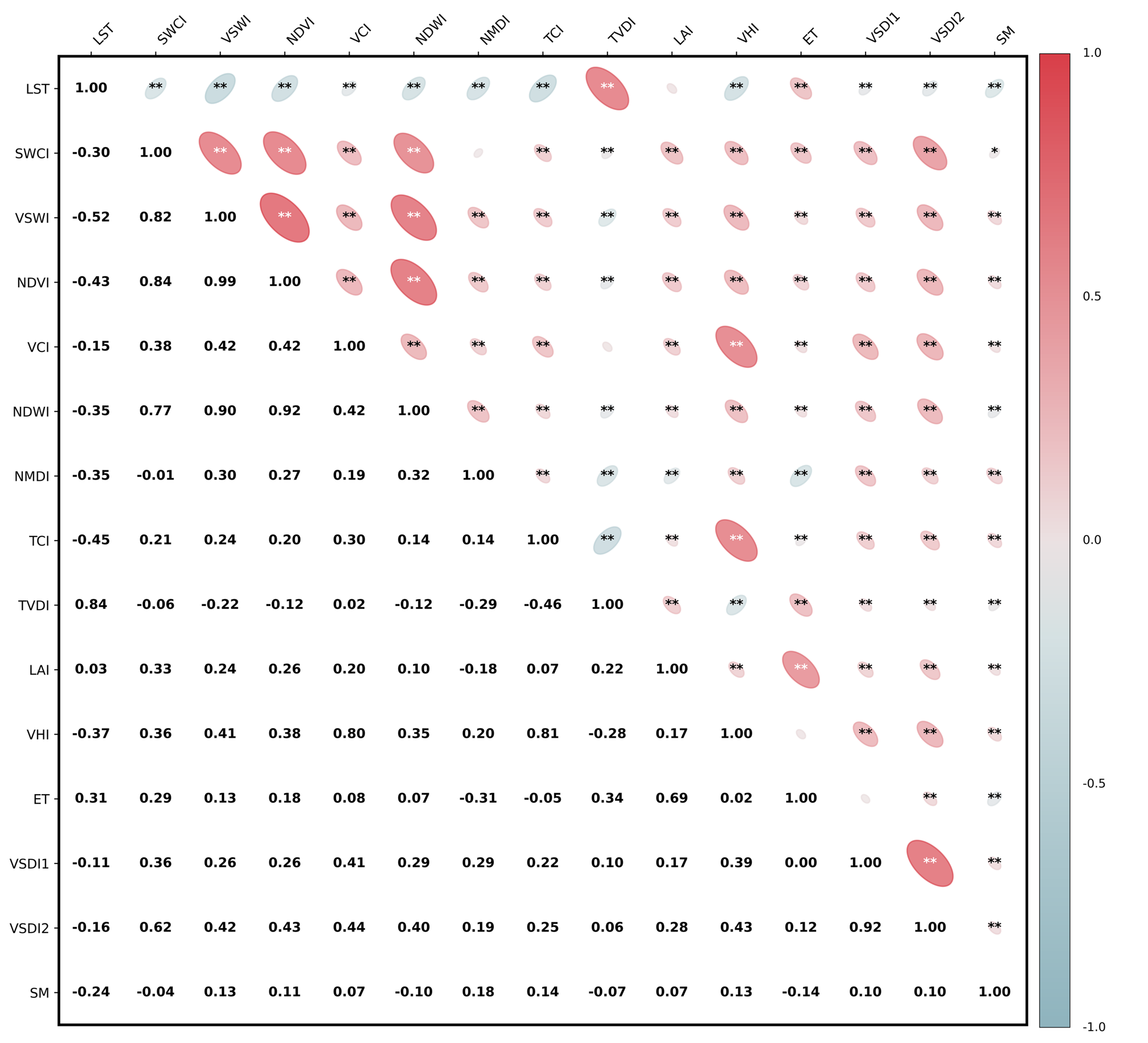

Supplement: S1 Fig — ** and * indicate significance levels at 1% and 5%, respectively. (PDF) [file pone.0351643.s001.pdf]

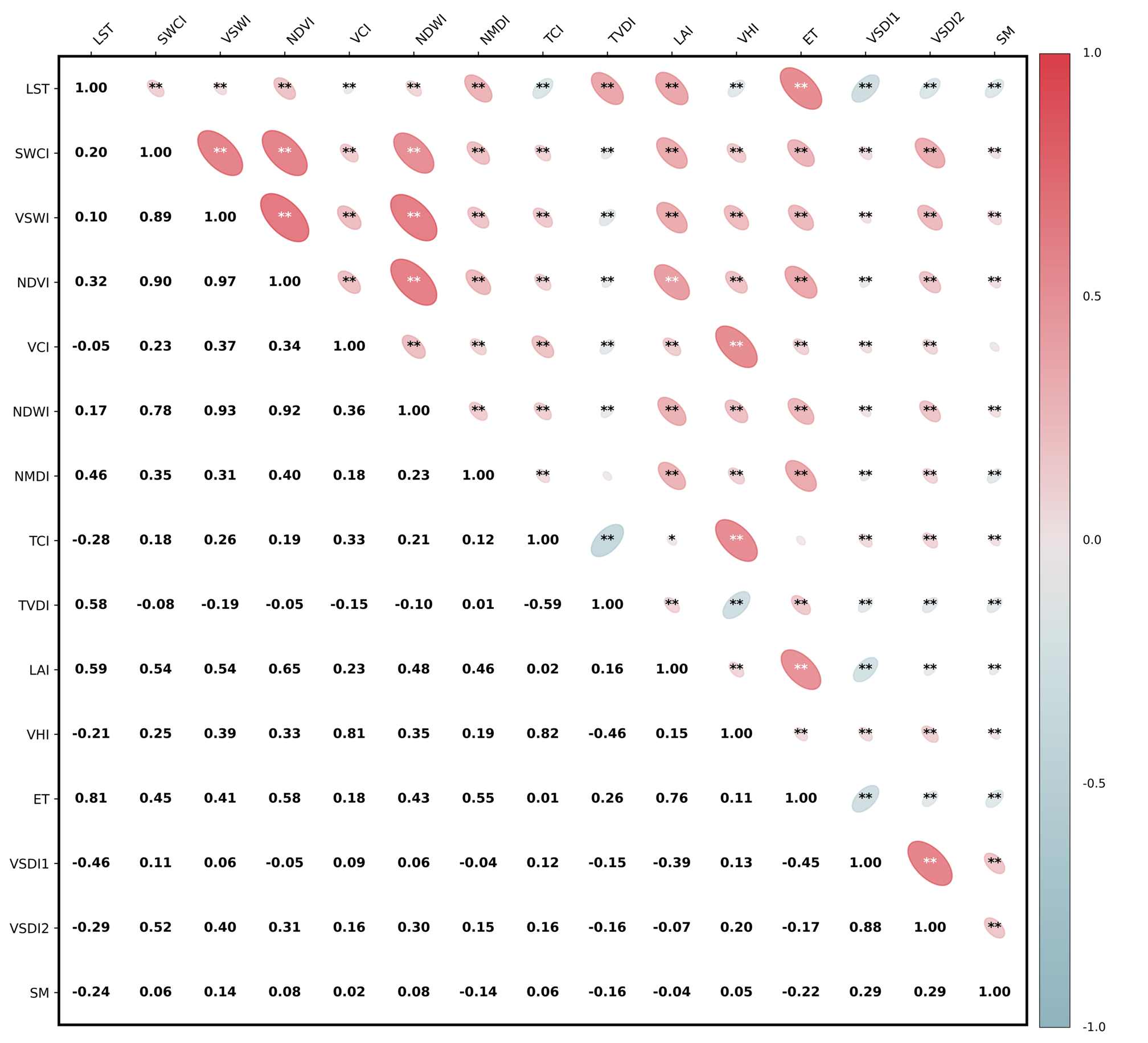

Supplement: S2 Fig — ** and * indicate significance levels at 1% and 5%, respectively. (PDF) [file pone.0351643.s002.pdf]

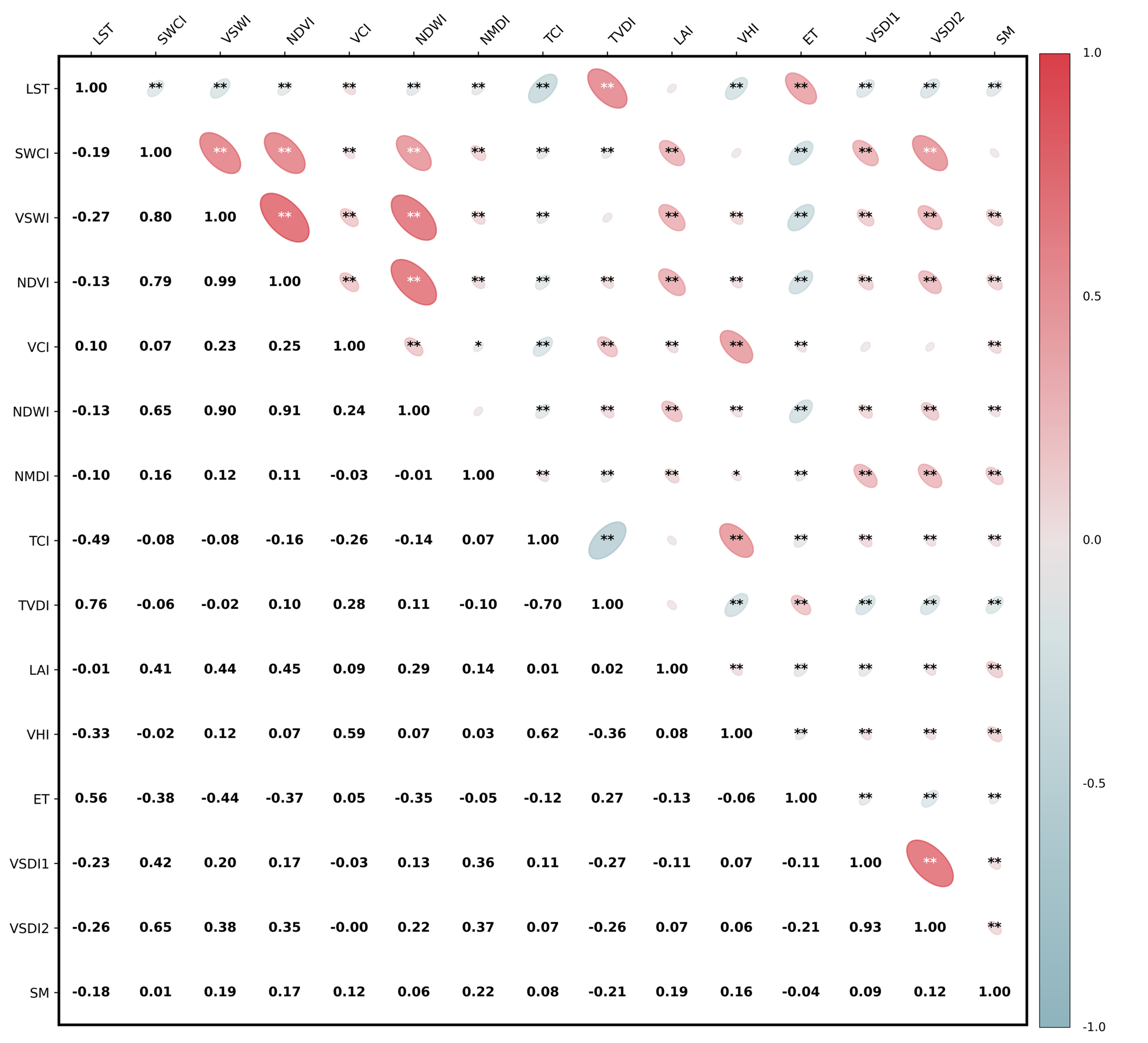

Supplement: S3 Fig — ** and * indicate significance levels at 1% and 5%, respectively. (PDF) [file pone.0351643.s003.pdf]
