# Supplementary material for: Machine learning-based seasonal SMAP soil moisture retrieval integrating MODIS drought indices: A case study of the Wujiang River Basin
Source: PLoS One. 2026 Jun 22;21(6):e0351643. doi: 10.1371/journal.pone.0351643 (PMC13286200; doi:10.1371/journal.pone.0351643)
Supplement: S7 Fig — (PDF) [file pone.0351643.s007.pdf]

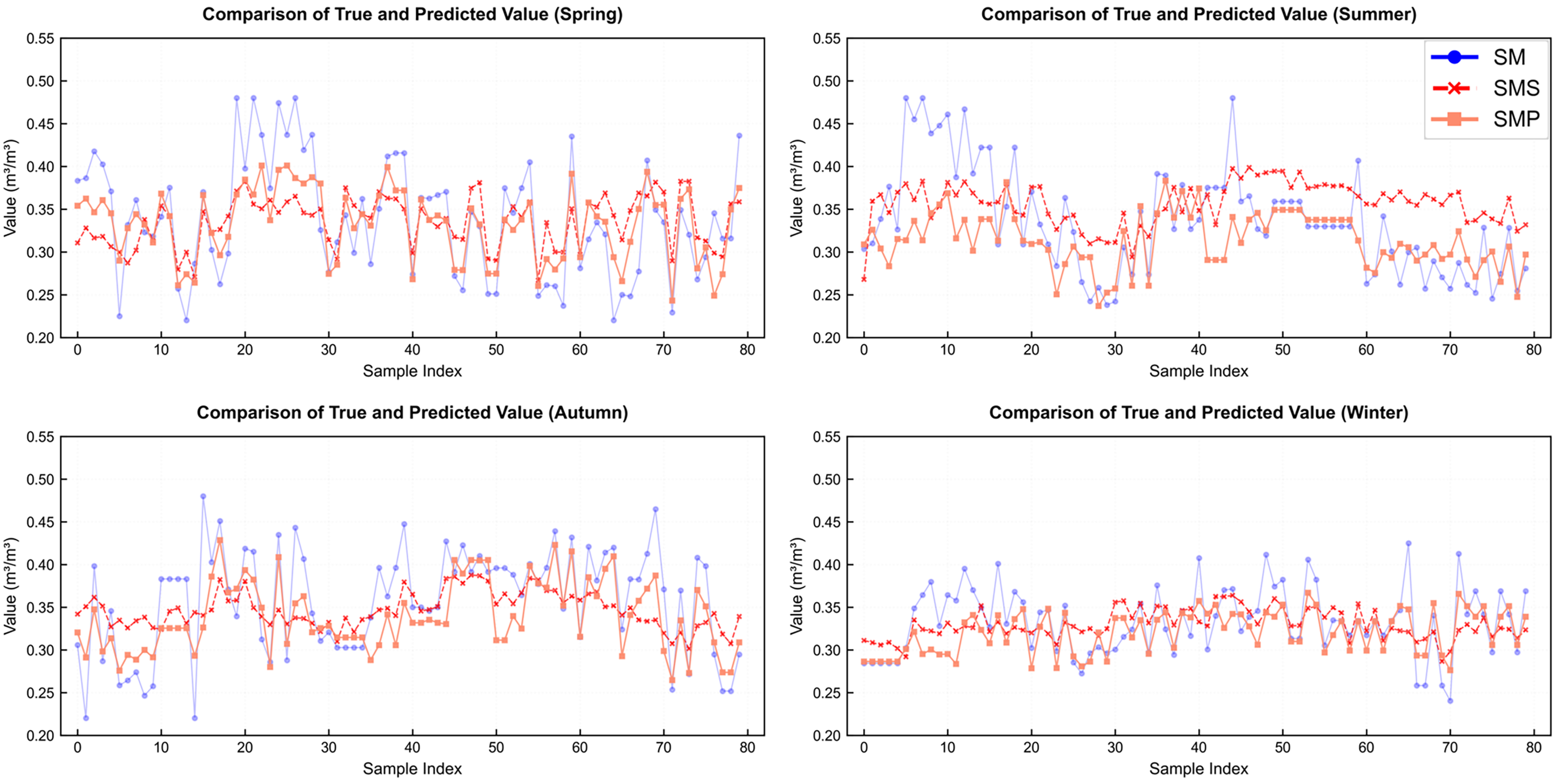

Comparison of True and Predicted Value (Spring)
Comparison of True and Predicted Value (Summer)
Comparison of True and Predicted Value (Autumn)
Comparison of True and Predicted Value (Winter)
Value (m³/m³)
Sample Index
SM
SMS
SMP
0.55
0.50
0.45
0.40
0.35
0.30
0.25
0.20
0
10
20
30
40
50
60
70
80
