# Supplementary material for: Machine learning-based seasonal SMAP soil moisture retrieval integrating MODIS drought indices: A case study of the Wujiang River Basin
Source: PLoS One. 2026 Jun 22;21(6):e0351643. doi: 10.1371/journal.pone.0351643 (PMC13286200; doi:10.1371/journal.pone.0351643)
Supplement: S1 Appendix — (DOCX) [file pone.0351643.s009.docx]

**S1 Appendix. Seasonal optimal hyperparameters for comparative machine learning models.**

**Table A. Optimal hyperparameter combinations for XGBoost, Random Forest (RF), Support Vector Machine (SVM), and RBFNN models.**

| Model | Season | Optimal Hyperparameters |
| --- | --- | --- |
| XGBoost | Spring | n :761, D :4, LR :0.036, γ :0.001, α :0.053 |
|  | Summer | n :1373, D :4, LR :0.022, γ :0.013, α :0.066 |
|  | Autumn | n :1328, D :6, LR :0.064, γ :0.046, α :0.084 |
|  | Winter | n :1323, D :4, LR :0.018, γ :0.002, α :0.063 |
| RF | Spring | n :403, D :26, S :47, L :7, F :0.74 |
|  | Summer | n :200, D :26, S :48, L :19, F :0.73 |
|  | Autumn | n :769, D :29, S :35, L :11, F :0.77 |
|  | Winter | n :331, D :10, S :46, L :10, F :0.50 |
| SVM | Spring | C :88.34, γ :0.018, ε :0.028 |
|  | Summer | C :0.25, γ :0.059, ε :0.014 |
|  | Autumn | C :2.81, γ :0.052, ε :0.028 |
|  | Winter | C :46.39, γ :0.006, ε :0.025 |
| RBFNN | Spring | Cts :3750, Sd :0.74, λ : 4e-05 |
|  | Summer | Cts :2550, Sd :0.47, λ : 0.32 |
|  | Autumn | Cts :4350, Sd :0.62, λ : 8e-04 |
|  | Winter | Cts :3100, Sd :1.56, λ : 2e-08 |

Note: The optimal hyperparameters for each season are listed in the sequence of Spring, Summer, Autumn, and Winter. All parameters were optimized via the Bayesian optimization algorithm within the Optuna framework. The parameter definitions are as follows: XGBoost: n, number of base learners; D, maximum depth; LR, learning rate;$\gamma$, minimum loss reduction;$\alpha$, L1 regularization term. RF: n, number of trees; D, maximum depth; S, minimum samples to split; L, minimum samples at leaf; F, fraction of features. SVM: C, penalty parameter;$\gamma$, kernel coefficient;, epsilon in the loss function. RBFNN: Cts, number of hidden layer centers; Sd, distribution width;$\lambda$, ridge regression regularization coefficient.
